# Prime editing-mediated microhomology enables efficient replacement of large DNA

**DOI:** 10.1093/nar/gkag626

**Published:** 2026-06-22

**Authors:** Yuyang Xie, Pan Li, Zhiyong He, Honglin Huang, Dingzhou Wu, Zhao Ma, Shenjiong Feng, Jiadong Ran, Kaixuan Hou, Fei Gao, Xuguang Du, Mario R Capecchi, Sen Wu

**Affiliations:** Frontiers Science Center for Molecular Design Breeding (MOE), State Key Laboratory of Animal Biotech Breeding, College of Biological Sciences, China Agricultural University, Beijing 100193, China; Xianghu Laboratory, Hangzhou 311231, China; Frontiers Science Center for Molecular Design Breeding (MOE), State Key Laboratory of Animal Biotech Breeding, College of Biological Sciences, China Agricultural University, Beijing 100193, China; Frontiers Science Center for Molecular Design Breeding (MOE), State Key Laboratory of Animal Biotech Breeding, College of Biological Sciences, China Agricultural University, Beijing 100193, China; Frontiers Science Center for Molecular Design Breeding (MOE), State Key Laboratory of Animal Biotech Breeding, College of Biological Sciences, China Agricultural University, Beijing 100193, China; Frontiers Science Center for Molecular Design Breeding (MOE), State Key Laboratory of Animal Biotech Breeding, College of Biological Sciences, China Agricultural University, Beijing 100193, China; Frontiers Science Center for Molecular Design Breeding (MOE), State Key Laboratory of Animal Biotech Breeding, College of Biological Sciences, China Agricultural University, Beijing 100193, China; Frontiers Science Center for Molecular Design Breeding (MOE), State Key Laboratory of Animal Biotech Breeding, College of Biological Sciences, China Agricultural University, Beijing 100193, China; Frontiers Science Center for Molecular Design Breeding (MOE), State Key Laboratory of Animal Biotech Breeding, College of Biological Sciences, China Agricultural University, Beijing 100193, China; Frontiers Science Center for Molecular Design Breeding (MOE), State Key Laboratory of Animal Biotech Breeding, College of Biological Sciences, China Agricultural University, Beijing 100193, China; Frontiers Science Center for Molecular Design Breeding (MOE), State Key Laboratory of Animal Biotech Breeding, College of Biological Sciences, China Agricultural University, Beijing 100193, China; Animal Science Research Office, Sanya Institute of China Agricultural University, Sanya 572024, China; Department of Human Genetics, University of Utah School of Medicine, Salt Lake City, UT 84112, United States; Frontiers Science Center for Molecular Design Breeding (MOE), State Key Laboratory of Animal Biotech Breeding, College of Biological Sciences, China Agricultural University, Beijing 100193, China; Animal Science Research Office, Sanya Institute of China Agricultural University, Sanya 572024, China

## Abstract

Precise and efficient replacement of large genomic DNA segments without inducing double-strand breaks (DSBs) remains a central challenge in genome engineering. Traditional homologous recombination relies on DSBs and long homologous arms, yet it remains inefficient, while recombinase or integrase systems suffer from residual sequences at integration sites. Prime editing (PE), limited by the processivity of reverse transcriptase, struggles to integrate large fragments (>100 bp). To address this challenge, we introduce Prime Editing–Microhomology-Enabled Replacement (PREMIER), a DSB-free platform by installing single-stranded microhomology arms at donor and genomic junctions via PE. In cell lines, PREMIER achieved a mean efficiency of 63.4% (median 65.2%) in diverse target sites, with peak efficiencies reaching 85.9%, exceeding homology-directed repair by 10–20-fold and reducing off-target integrations by over 100-fold compared to nonhomologous end joining. It bypasses the need for long homology arms, simplifies donor preparation, achieves targeted replacement of sequences up to 10.3 kb. *In vivo*, PREMIER integrates a 6.2-kb oncogene cassette into the mouse liver. Additionally, PREMIER replaces murine *Trp53* with human *TP53* CDS, generating functional humanized mice. Altogether, PREMIER provides a precise, high-efficiency, and DSB-free strategy for large-scale genome rewriting, offering a powerful tool for complex modeling and therapeutic genome editing.

## Graphical Abstract

**Graphical Abstract:**
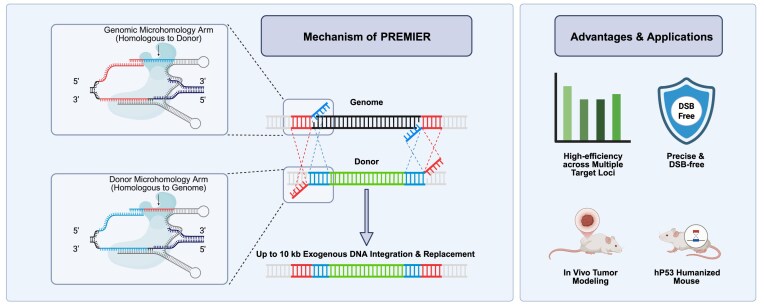

## Introduction

Precise integration and replacement of large DNA sequences (>1 kb) in mammalian genomes holds transformative potential for gene therapy, synthetic biology, and disease modeling. While CRISPR-Cas systems have revolutionized genome editing [1, 2], current approaches to large-fragment manipulation are hindered by intrinsic limitations. Homology-directed repair (HDR), the gold standard for precise integration, requires double-strand breaks (DSBs), and long homology arms (>300 bp) [3], yet it suffers from low efficiency (<5% in most cell types), cell cycle dependency [4, 5], and substantial DNA damage that compromises cell viability [6]. Concurrently, the requirement for long homology arms poses significant challenges for the delivery of donor constructs. Recent efforts to enhance large-fragment integration have focused on using small molecules to inhibit specific repair pathways to increase the capacity of HDR [7–10], which has greatly enhanced the integration efficiency of HDR. However, this approach may exhibit cytotoxicity and poses potential risks to genomic stability. Nonhomologous end joining (NHEJ) [11], although efficient, introduces unpredictable indels and widespread off-target integration [12–14]. Other approaches have explored microhomology-mediated mechanisms, such as microhomology-mediated end joining (MMEJ) [15–20] and single-strand annealing (SSA) [21, 22], which improve efficiency but still rely on DSBs and carry risks of chromosomal rearrangements, or involve overly complex donor preparation processes.

Prime editing (PE) [23], a more recent innovation, enables DSB-free editing of small sequences but fails to accommodate large-fragment integration (>100 bp) due to the limited processivity of reverse transcriptase (RT) [24, 25]. A recent strategy called PAINT has demonstrated the potential for integrating larger exogenous sequences by introducing microhomology arms via PE, which facilitates HDR [26]. However, this strategy necessitates the generation of DSBs and the use of two distinct Cas9 proteins, thereby complicating the process and potentially introducing additional genomic instability.

Transposon- and integrase-based methods bypass DSBs but often lack programmability and leave behind residual sequences at the integration site [27, 28]. Attempts to combine PE with recombinase systems (e.g. Bxb1-attB/attP) [29–32] or CRISPR-Cas with transposase systems [33] enable targeted insertion but leave large genomic scars. Conversely, the integration of retrotransposons with CRISPR-Cas9 nickase offers scarless integration, however, its efficiency is relatively low [34]. While these advances represent important progress in enhancing genome-editing precision and efficiency, challenges remain in balancing precision, efficiency, and avoiding DSB-associated genotoxicity. Our approach aims to further improve upon these foundations. Thus, a generalizable method for efficient, precise, DSB-free, and long-homology-arm-independent replacement of multi-kilobase genomic sequences remains an unmet need.

To address this, we developed Prime Editing–Microhomology-Enabled Replacement (PREMIER), a genome editing strategy that leverages PE to generate four single-stranded microhomology arms (20∼60 nt) at the junctions of both genomic target sites and donor constructs. This approach enables seamless and efficient replacement of DNA segments up to 10.3 kb without the need for long homology arms or introducing DSBs. PREMIER achieves markedly higher efficiency than HDR while maintaining high fidelity and drastically reducing off-target effects associated with NHEJ. Systematic optimization of RT activity, prime editing guide RNA (pegRNA) architecture, and donor design further enhanced integration efficiency.

We demonstrate the versatility and robustness of PREMIER across diverse loci and cell types. Moreover, PREMIER facilitates multiplexed editing and shows strong translational potential: it enabled *in vivo* liver tumor modeling via integration of a triple-oncogene cassette and supported the generation of functional humanized *TP53* chimeric mice through precise replacement of murine *Trp53*. Collectively, PREMIER establishes a new framework for large-fragment genome rewriting that combines precision, scalability, and minimal genotoxic stress, paving the way for advanced genetic engineering applications.

## Materials and methods

### Ethics statement

All animal experiments described in this study were conducted with the approval of the Animal Welfare Committee of China Agricultural University (approval no. AW03111202-3-1) and were performed in accordance with relevant institutional guidelines for the care and use of laboratory animals. Mice were housed in a specific pathogen-free facility under controlled environmental conditions, with temperature and humidity maintained at constant levels. Animals were maintained on a 12 h light/12 h dark cycle, with lights on from 6:00 a.m. to 6:00 p.m., and had access to food and water ad libitum. The physical condition and behavioral status of the mice were monitored regularly throughout the study. Humane endpoint criteria included significant body weight loss, poor general condition, abnormal posture or appearance, markedly reduced activity, impaired mobility, or other signs of distress or suffering. Animals meeting these predefined humane endpoint criteria were euthanized promptly to minimize pain and distress. At the end of the study, mice were humanely euthanized by cervical dislocation. All efforts were made to minimize animal suffering and to reduce the number of animals used.

### Plasmids construction

The coding sequences (CDS) of various RT fragments used in this study were synthesized by Genewiz. The N-terminal domain of the RNA-binding exonuclease protection factor La was obtained from HEK293T complementary DNA (cDNA) via polymerase chain reaction (PCR). The gRNA expression vectors were constructed by annealing oligonucleotides, which were subsequently ligated to the pCRISPR-sg6 backbone digested with Bbs1-HF (NEB, R3539M) using T4 ligase (NEB, M0202L). For pegRNA expression vectors, oligonucleotides were generated by phosphorylation of three pairs of primers using the T4 PNK Kit (Beyotime, D7097), followed by gradient annealing. These primers annealed to form gRNA, scaffold, and reverse transcription template (RTT) + primer binding site (PBS). For engineered prime editing guide RNA (epegRNA), an additional pair of primers was used to form the tevopreQ1 domain. The resulting oligonucleotides were then ligated to the pCRISPR-sg6 backbone, digested with Bbs1-HF (NEB, R3539M) using T4 ligase (NEB, M0202L). PCR amplification was performed using the Q5^®^ High-Fidelity PCR Kit (NEB, E0555L). DNA fragments were purified using the FastPure Gel DNA Extraction Mini Kit (Vazyme, DC301-01). The donor plasmids and plasmids expressing Cas9 and RT were constructed using the GenRec Assembly Master Mix Kit (General Biol, CL08050). Plasmids expressing pegRNA in tandem were assembled using the NEBridge^®^ Golden Gate Assembly Kit (BsaI-HF^®^v2, E1601). All plasmids used in this study were endotoxin-free and purified using the Endo-Free Plasmid Mini Kit I (Omega, D6948-02) and confirmed by Sanger sequencing. All pegRNA and gRNA sequences used in this study are provided in Supplementary Table S1. All donor and protein expression vector sequences used in this study are provided in Supplementary Table S2.

### Cell line authentication

HEK293T, Hela, PK15 cell lines used in this study were obtained from the American Type Culture Collection (ATCC), these cell lines were authenticated by ATCC and not further validated in our laboratory. The mouse embryonic stem cells (mESCs) were derived from male embryos of 129 × C57/BL6 mouse hybrids and were authenticated by our laboratory. All cell lines used were tested for mycoplasma contamination and were found to be free of mycoplasma.

### Cell culture

HEK293T, Hela cells were kept in Dulbecco’s modified Eagle medium (DMEM) containing L-glutamine and sodium pyruvate (Servicebio, G4520-500ML), supplemented with 10% fetal bovine serum (FBS, Gibco, 10 099), 1% nonessential amino acids (Gibco, 11 140 050), and 1% penicillin/streptomycin (Gibco, 15 104 122). Mouse ESCs were maintained on feeder cells using a basic serum/leukemia inhibitory factor (LIF) medium. This medium comprised DMEM (Gibco, 10 829 018), 15% FBS (Gibco, 10 099), 1% penicillin/streptomycin (Gibco, 15 104 122), 1% nonessential amino acids (Gibco, 11 140 050), 1% GlutaMAX (Gibco, 35 050 079), 106 units/l of mouse LIF (Millipore, ESG1106), and 100 mM β-mercaptoethanol (Gibco, 15 104 122). The feeder cells were treated with mitomycin C (Amresco, MJ594) to inhibit their growth and were cultured in DMEM (Gibco, 11 960) supplemented with 10% FBS (Gibco, 10 099), 1% penicillin/streptomycin (Gibco, 15 104 122), 1% nonessential amino acids (Gibco, 11 140 050) and 1% sodium pyruvate (Gibco, 15 104 122).

### Cell transfection

To determine the insertion efficiency of PREMIER, 1 × 10^5^ HeLa cells were seeded per well in a 24-well plate. After 24 h, cells were transfected with LentiFit™ (Hanbio, Let1000) according to the manufacturer’s instructions. For each well of the 24-well plate, a total of 0.6 µg of the plasmid expressing Cas9 and RT, 0.2 µg of the donor plasmid, and four pegRNA expression plasmids (0.15 µg each) were co-transfected. Cells were incubated at 37°C for 8 h, after which the medium was replaced with fresh medium. At 24 h post-transfection, the medium was replaced with fresh medium containing puromycin (1 µg/ml) for selection of transfected cells. The selection was maintained for 48 h, after which the cells were returned to fresh medium without puromycin. For cells transfected with donor plasmids expressing fluorescent proteins, cells were passaged according to density and cultured for 14 days to allow for the complete metabolism of the transiently expressed fluorescent proteins. The integration efficiency was then assessed by flow cytometry, measuring the proportion of cells retaining green fluorescence. For cells transfected with donor plasmids containing an internal ribosome entry site (IRES) or a 2A peptide fusion upstream of the fluorescent protein, the integration efficiency was assessed by flow cytometry 48 h after the completion of puromycin selection, measuring the proportion of cells expressing green fluorescence.

### High-throughput DNA sequencing of endogenous sites and data analysis

Fifty thousand fluorescence-positive cells, collected by flow cytometry sorting, were centrifuged at 300 × *g* for 5 min. The supernatant was removed, and the cells were resuspended in 30 µl of lysis buffer supplemented with 1 µl of proteinase (Vazyme, PD101-01) in a PCR tube. The mixture was incubated at 55°Cfor 2 h, followed by 95°C for 15 min. The resulting genomic DNA was stored at −20°C. The junction sequences between the inserted exogenous sequences and the endogenous sequences were amplified using 2 × Taq Master Mix (Vazyme, P112). One primer was designed to bind to the endogenous site, while the other bound to the inserted exogenous sequence. The primers incorporated adapter sequences: the forward primer was 5′-CTGCAAGGCGATTAAGTTGGGTA-3′, and the reverse primer was 5′-GCAGTGAGCGCAACGCAATT-3′. All primer sequences used in this study are provided in Supplementary Table S3. The first-round PCR products were subjected to a second round of PCR amplification, using primers carrying distinct barcode sequences to differentiate samples. The products from the second-round PCR were pooled to form a library, which was purified using the FastPure Gel DNA Extraction Mini Kit (Vazyme, DC301-01). The purified products were then sequenced on the Illumina MiSeq platform. The obtained data were processed and analyzed using CRISPResso2 [35]. Reads with an average quality score below 15 and single-base quality scores below 3 were filtered out. The minimum paired-end read overlap was set to 5 bp.

### Genome-wide off-target integration analysis by Tn5 transposome and next-generation sequencing

To quantify off-target integrations induced by PREMIER and NHEJ across the human genome, cells were collected 14 days post-staining. Genomic DNA was extracted and purified according to the manufacturer’s protocol using the FastPure DNA Isolation Mini Kit (Vazyme, DC112). Transposase (Tn5, 145ES8) and Adapter Mix were used to tag the genome, followed by shearing and purification of 200–400 bp DNA fragments using VAHTS DNA Clean Beads (Vazyme, N411-01). Library construction was performed following the VAHTS Universal DNA Library Prep Kit for Illumina NovaSeq 6000 (Vazyme, ND607-01) manufacturer’s instructions. Quality control of the libraries was assessed using the Agilent 2100, and paired-end 150 bp sequencing (PE150) was conducted on the Illumina NovaSeq 6000 platform. Raw sequencing data were quality-controlled using Fastp and aligned to the hg38 reference genome using bowtie2. Integration events in the genome were accurately identified through an integrated site-specific analysis pipeline. Finally, bioinformatics analysis was performed to calculate the genome-wide off-target integration frequency.

### RNA extraction and qPCR

Total RNA was extracted from cells using the RaPure Total RNA Mini Kit (Magene, R4011) following the manufacturer’s protocol. Subsequently, RNA was reverse-transcribed into cDNA using the ABScript III Reverse Mix for quantitative polymerase chain reaction (qPCR) with gDNA Remover (Abclonal, RK20429). qPCR was performed using the 2×Universal SYBRGreen Fast qPCR Mix (Abclonal, RK21203) on the Light Cycler^®^ 96 Instrument.

Relative genomic copy number was determined by qPCR using the 2^−ΔΔCt^ method. Specific primer pairs were designed to amplify either the insertion cargo or the replaced endogenous sequence at the *PPP1R12C* locus (primer sequences are provided in Supplementary Table S3). *B2M* was used as reference gene for normalization.

### Hydrodynamic tail vein injection

The experimental subjects were 4-week-old male ICR mice, weighing between 28 and 30 g, obtained from Beijing Vital River Laboratory Animal Technology Co., Ltd., a subsidiary of Charles River Laboratories. Prior to the surgical procedure, a water bag was heated to 55°C to facilitate dilation of the tail veins. Plasmids were diluted to the appropriate concentration with saline, with an injection volume of 3 ml per mouse. For each experimental group of mice, the plasmid doses administered were as follows: nCas9-RT 38 µg, donor plasmid expressing three oncogenes in tandem 12 µg, and plasmids expressing four pegRNAs targeting the donor and endogenous sites in parallel 10 µg. A 5 ml syringe was used to draw the plasmid solution, fitted with a 1 ml syringe needle for the injection. Mice were restrained in a tail vein injection holder, with their tails carefully straightened and tautened. Post-injection monitoring was essential, as this method carried a certain risk of mortality. The resumption of feeding and drinking by the mice ∼6 h post-injection indicated a successful tail vein injection.

### Immunofluorescence

Cells were initially fixed with a 4% paraformaldehyde solution for 30 min to preserve their structural integrity. Subsequently, cells were permeabilized with 0.5% Triton X-100 for 30 min at room temperature to facilitate antibody penetration. To minimize nonspecific binding, cells were blocked with Immunol Staining Blocking Buffer (Beyotime, P0102). Cells were then incubated with primary antibodies against KI67 (Beyotime, C2301S, 1:50) and γ-H2A.X (Beyotime, C2037S, 1:50) overnight at 4°C, followed by incubation with secondary antibodies (1:500) for 1 h at room temperature. Cell nuclei were counterstained with DAPI (Beyotime, P0131) to visualize their morphology. The samples were examined and imaged using a fluorescence microscope.

### Tissue and cell protein extraction and western blotting

Tissue pieces and cell pellets were resuspended in an appropriate amount of IP lysis buffer (HuaxingBio, HX1798) supplemented with phenylmethylsulfonyl fluoride (PMSF) (100×). For tissues, a homogenizer was used to create a uniform suspension, while for cells, the pellet was thoroughly mixed by pipetting and then incubated on ice for 30 min. Following this, the mixture was centrifuged at 4°C, 13 000 × *g* for 10 min, and the supernatant, containing the protein solution, was collected and stored at −80°C.

For Western blotting, 40 µl of protein solution was mixed with 10 µl of 5 × sodium dodecyl sulphate–polyacrylamide gel electrophoresis Sample Loading Buffer and denatured at 95°C for 10 min. Samples were then loaded onto a 4%–20% Precast Protein Plus Gel (Yeasen, 36270ES10), with a 180 kDa Prestained Protein Marker (Vazyme, MP102) serving as a molecular weight standard. Electrophoresis was conducted at 80 V for 1 h, followed by 120 V for 30 min. Proteins were transferred to a polyvinylidene difluoride (PVDF) membrane at 400 mA for 30 min. The membrane was then blocked with NcmBlot Blocking Buffer (NCM Biotech, P30500) for 15 min on a shaker at room temperature. After blocking, the membrane was washed three times with Tris-buffered saline with Tween 20 (TBST) for 5 min each, followed by incubation with the primary antibody overnight at 4°C. The primary antibody was removed, and the membrane was washed again with TBST three times for 5 min each, before incubation with the secondary antibody for 1 h at room temperature. The membrane was then washed three times with TBST for 5 min each, and finally, the proteins were visualized.

### Histological analysis

The mouse liver tissues were collected and fixed in a 4% paraformaldehyde solution for a duration of 2–4 h. Following fixation, the tissues were subjected to a series of graded ethanol washes for dehydration and then embedded in paraffin. Paraffin-embedded tissues were sectioned into 5 μm slices in preparation for hematoxylin and eosin (H&E) staining. The sections were first deparaffinized with xylene and rehydrated through a descending series of ethanol concentrations to facilitate dye penetration. Hematoxylin staining was applied to the section, which was followed by eosin counterstaining. The stained sections were then dehydrated using an ascending series of ethanol concentrations and xylene, and finally, the slides were mounted with neutral gum to preserve and secure the sections for microscopic examination.

### Statistical analysis

All statistical analyses were performed using GraphPad Prism 9 or R software. Data are presented as mean ± standard deviation (SD) unless otherwise stated. The number of independent biological replicates (n) is indicated in the figure legends.

For comparisons between two groups, two-tailed unpaired Student’s *t*-test was used. For multiple group comparisons, one-way analysis of variance (ANOVA) was applied. ns, not significant; *P <.05; **P <.01; ***P <.001; ****P <.0001. All experiments were repeated at least three times independently with similar results.

## Results

### PREMIER enables DSB-free and efficient integration of large DNA sequences *in vitro*

**Figure 1.**
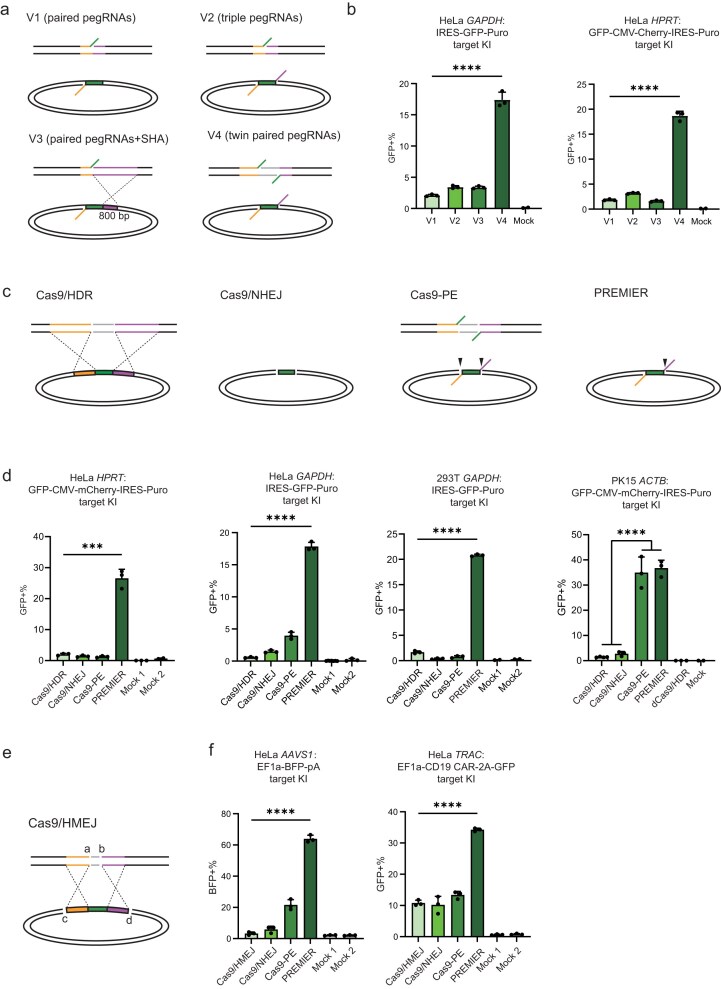
PREMIER allows efficient targeted integration of exogenous sequences without inducing DSBs. (**a**) Schematic diagram showing four PE-mediated knock-in strategies. Each strategy uses PE2 to generate single-stranded micro-homologous flaps at donor and genomic sites. V1 uses one pegRNA pair for flaps mediating foreign DNA integration. V2 adds a pegRNA in the donor vector for downstream flaps. V3 appends an 800 bp homology arm downstream of the nick site. V4 employs two pegRNA pairs for four flaps for targeted integration. (**b**) Integration efficiency of a fluorescent reporter system at the *GAPDH* and *HPRT* loci mediated by four distinct PE-mediated knock-in strategies (V1, V2, V3, V4). (**c**) Schematic diagram shows HDR-, NHEJ-, Cas9-PE, and PREMIER-mediated targeted integration of foreign DNAs. HDR requires two adjacent DSBs at the genomic target site and two 1000 bp homologous arms. NHEJ needs two adjacent DSBs in the targeted genomic locus and cleave the transgene cassette. Cas9-PE and PREMIER generate single-stranded micro-homologous flaps at both ends of the transgene cassette and the two adjacent genomic target sites; Cas9-PE introduces DSBs through Cas9-PE/pegRNA system, while PREMIER introduces nicks. (**d**) Integration efficiency of a fluorescent reporter system at various endogenous loci mediated by PREMIER, Cas9-PE, HDR, and NHEJ in several cell types. (**e**) Schematic diagram shows HMEJ-mediated targeted integration of foreign DNAs, HMEJ-based approach required the introduction of two adjacent DSBs at the genomic target site, as well as two DSBs at both ends of the donor DNA using Cas9, along with the donor vector which has ∼800 bp of the homologous arm. (**f**) Integration efficiency of a fluorescent reporter system at various endogenous loci in HeLa cells mediated by PREMIER, Cas9-PE, HMEJ, and NHEJ. Data are presented as means ± SD from *n* = 3 independent biological replicates. Statistical significance was assessed using Student’s *t*-test. ns, not significant; *P <.05; **P <.01; ***P <.001; ****P <.0001.

To establish a DSB-free method for large-fragment integration without using long homology arms, we first systematically evaluated four distinct PE-dependent knock-in strategies (Fig. 1A), each of which version utilized PE2 to generate single-stranded microhomologous flaps. In version V1, a single pair of pegRNAs generated complementary microhomology arms, each 35 bp in length, mediating foreign DNA integration. The single-stranded DNA (ssDNA) at the genomic target site is homologous to the upstream sequences of the nick site in the donor vector, and conversely, the ssDNA at the donor vector’s target site is homologous to the upstream sequences of the nick site in the genomic target site. Version 2 (V2) incorporated an additional pegRNA within the donor vector to produce a flap homologous to sequences downstream of the genomic nick site. Version 3 (V3) appended an 800 bp homology arm downstream of the insert in the donor, homologous to genomic sequences distal to the nick in the genome site. Version 4 (V4) employed two independent pairs of pegRNAs, generating microhomology arms with a length of 35 bp at both ends of the replacement segment in the genome and the donor insert, which were like the paired pegRNAs used in Version 1. Then the two ssDNA flaps in the separated two target genomic loci were designed to be homologous to the DNA sequences of integration. And the ssDNA flaps on both sides of the part of integration in the donor vector were homologous to the upstream of the targeted nick sites in the genome. In the design of the pegRNAs for the experiment, we set the primer binding site (PBS) annealing temperature to ∼40°C, as this has been proved to be an optimal annealing temperature for mammalian cells [36]. Quantitative assessment at the *HPRT* and *GAPDH* loci in HeLa cells using flow cytometry for GFP-positive cells post-puromycin selection revealed V4 as the optimal configuration, achieving peak integration efficiencies of 18.7% at *HPRT* and 17.4% at *GAPDH* (Fig. 1B), Sanger sequencing of the junctions between the integrated fragments and the genomic DNA revealed correct sequences with no mixed peaks (Supplementary Fig. S1A). This represented a significant improvement over V1–V3, demonstrating that bidirectional microhomology arm generation via PE effectively substitutes traditional homology arms. We designated this optimized strategy PREMIER.

We next performed a comprehensive comparative analysis of PREMIER against established genome-editing methodologies—HDR, NHEJ, HMEJ [37], and a Cas9-PE-based approach, which is a variant of PREMIER where the nCas9-RT is replaced with Cas9-RT, while other components remain unchanged. The comparative analysis was carried out across multiple genomic loci (*HPRT, GAPDH, ACTB, AAVS1, TRAC*) and diverse cell types (HeLa, HEK293T, PK15). Two reporter systems were employed: (i) a promoterless GFP cassette with cytomegalovirus (CMV) promoter-driven mCherry-IRES-Puro, and (ii) an IRES-GFP cassette with SV40-Puro, both reporter systems require integration into endogenous loci to enable expression of GFP under the control of the endogenous gene promoters (Supplementary Fig. S1B). Following transfection, puromycin selection (48 h) was employed to isolate transfected cells, and a 7-day culture period, fluorescence-activated cell sorting analysis quantified EGFP + cells as a direct measure of knock-in efficiency. PREMIER consistently outperformed all comparator methods. At the *HPRT* and *GAPDH* loci, PREMIER efficiency significantly exceeded HDR, NHEJ, and Cas9-PE in both HeLa and HEK293T cells. In PK15 cells targeting *ACTB*, PREMIER, and Cas9-PE demonstrated comparable efficiency, both markedly superior to HDR and NHEJ (Fig. 1D). As the HMEJ-based strategy improved knock-in efficiency compared to the HDR-based method, we then examined whether the PREMIER-based method showed a more robust knock-in efficiency compared with the HMEJ-based method. When targeting the *AAVS1* locus (EF1a-BFP reporter) and the *TRAC* locus (EF1a-CD19 CAR-2A-GFP reporter) in HeLa cells, PREMIER exhibited substantially higher integration efficiency than HMEJ, NHEJ, and Cas9-PE (Fig. 1F), the integration events were also confirmed by amplifying the junctions and sequencing (Supplementary Fig. S1C and D). These results establish PREMIER as a robust and versatile platform for precise, DSB-free integration, demonstrating superior efficiency across varied experimental contexts.

### Systematic optimization enhances PREMIER efficiency

**Figure 2.**
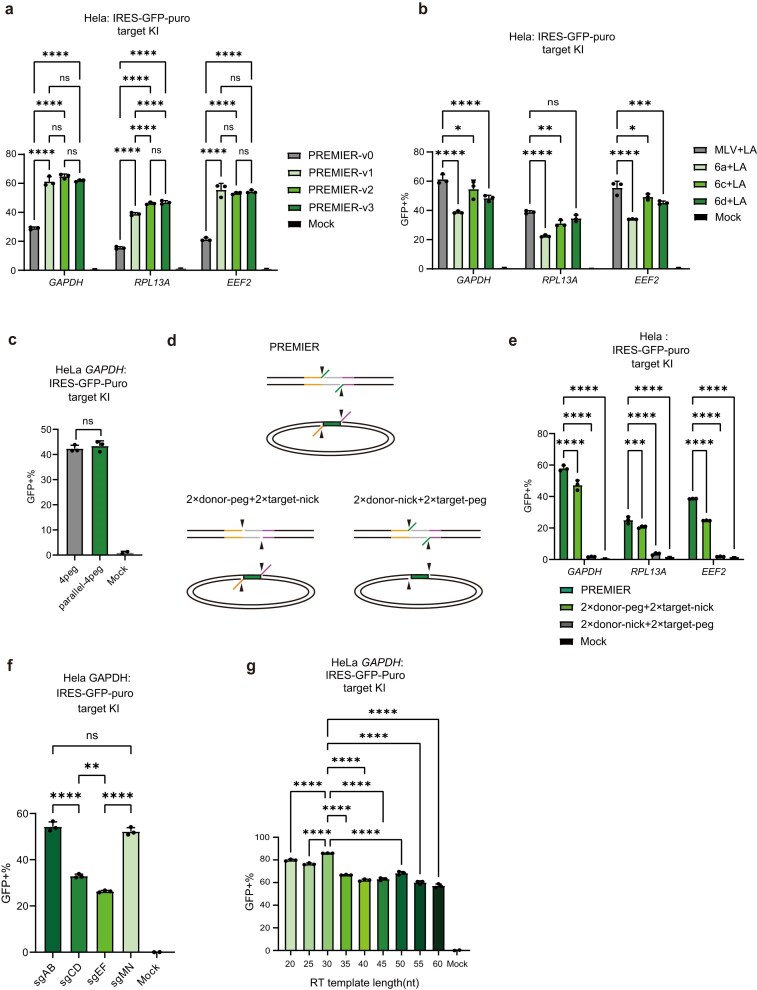
Optimization of PREMIER-Mediated Exogenous Sequence Integration. (**a**) Integration efficiency of IRES-GFP at the *GAPDH, RPL13A*, and *EEF2* loci in HeLa cells mediated by four distinct PREMIER strategies: PREMIER-v0, PREMIER-v1, PREMIER-v2, and PREMIER-v3. *n* = 3 independent biological replicates. (**b**) Efficiency of IRES-GFP integration at the *GAPDH, RPL13A*, and *EEF2* loci in HeLa cells using PREMIER-v1 strategy with MLV-RT and three truncated RTs (PE6a, PE6c, PE6d). (**c**) Comparison of integration replacement efficiency of IRES-GFP at the *GAPDH* locus in HeLa cells when four pegRNAs are expressed from separate vectors versus co-expressed from a single vector. Statistical significance was determined using *t*-tests. ns, no significance. (**d**) Diagram illustrating the scenario where two pegRNAs targeting the genome are replaced with gRNAs while retaining two pegRNAs targeting the donor (2 × donor-peg + 2 × target-nick), and vice versa (2 × donor-nick + 2 × target-peg), showing the microhomology arms and nicks at the donor and target sites. The triangular markers denote the positions of single-strand nicks. (**e**) Integration efficiency of IRES-GFP at the *GAPDH, RPL13A*, and *EEF2* loci in HeLa cells mediated by three different strategies: 2 × donor-peg + 2 × target-nick, and 2 × donor-nick + 2 × target-peg. (**f**) Integration efficiency of IRES-GFP at the *GAPDH* locus in HeLa cells mediated by PREMIER with four different pegRNA target sequences at both ends (sgAB, sgCD, sgEF, sgMN). (**g**) Integration efficiency of IRES-GFP at the *GAPDH* locus in HeLa cells by PREMIER with varying lengths of the RTT in pegRNAs. *n* = 3 independent biological replicates. Data are represented as means ± SD. Statistical significance was determined using one-way ANOVA. ns, no significance; *P <.05; **P <.01; ***P <.001; ****P <.0001.

To further enhance the efficiency and performance of PREMIER, as the stability of pegRNA has a marked influence on the editing efficiency of PE, we implemented two synergistic optimization strategies: (i) Fusion of the N-terminal RNA-binding domain of the exonuclease protection factor La [La (1–194)] [38] to the N-terminus of MLV-RT, termed PREMIER-v1; (ii) Replacement of standard pegRNA with engineered pegRNA (epegRNA) incorporating a tevopreQ1 stability domain [39], termed PREMIER-v2. Additionally, we combined the strategies above, termed PREMIER-v3 (Supplementary Fig. S3A). Assessment of these variants using an IRES-GFP reporter integrated into the *GAPDH, RPL13A*, and *EEF2* loci in HeLa cells (Supplementary Fig. S2) revealed that all optimized versions yielded at least a two-fold increase in efficiency compared to the baseline PREMIER, with peak efficiency reaching 64.87% (Fig. 2A). Statistical analysis indicated no significant difference in efficacy between the three optimization strategies. Considering potential complexities in epegRNA construction and computational predictions suggesting limited benefit from the tevopreQ1 linker in certain contexts, PREMIER-v1 (RT-La fusion) was selected for subsequent studies.

To address potential delivery constraints, we evaluated various truncated RTs identified in previous studies [40]. Notably, variants PE6c and PE6d mediated highly efficient integration (54.57% and 48.33%, respectively), comparable to full-length MLV-RT used in PE2max (Fig. 2B and Supplementary Fig. S3B). Furthermore, co-expression of all four required pegRNAs from a single plasmid via a tandem construct maintained high integration efficiency (Fig. 2C and Supplementary Fig. S3C), simplifying delivery logistics. This co-expression strategy was adopted for future applications.

To confirm the critical role of PE-generated microhomology arms, we replaced pegRNAs targeting either the genome or the donor with standard nickase gRNAs (lacking RTTs), which significantly impaired integration. Absence of genomic microhomology arms caused a moderate efficiency reduction, while absence of donor microhomology arms nearly abolished integration (Fig. 2E), underscoring the essential function of donor-targeting pegRNAs. Thus, we concluded that the targeting efficiency of pegRNAs at both ends of the donor has a considerable impact on the efficiency of PREMIER-mediated large fragment integration. Subsequently, we designed four distinct sequences for the pegRNA target sites at both ends of the donor, labeled as sgAB, sgCD, sgEF, and sgMN (Supplementary Fig. S3D). Optimization of pegRNA target site sequences within the donor vector identified sgAB and sgMN sequences as yielding significantly higher integration efficiency at the *GAPDH* locus compared to sgCD and sgEF (Fig. 2F), further demonstrating the substantial influence of the pegRNA efficiency at both ends of the donor on PREMIER’s integration efficiency. Finally, systematic variation of the RTT length (20–60 nt) revealed maximal integration efficiency (85.9%) at an RTT length of 30 nt (Fig. 2G and Supplementary Fig. S4).

### PREMIER facilitates large-scale replacement and multiplexed editing

**Figure 3.**
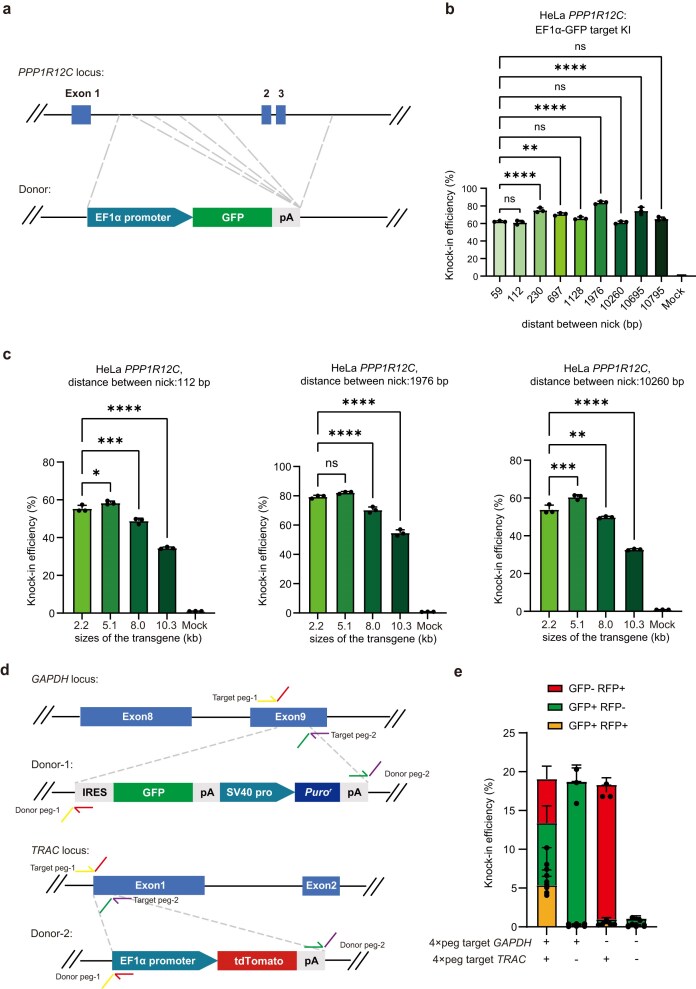
Assessment of PREMIER-Mediated Exogenous Sequence Integration. (**a**) Schematic representation of PREMIER-mediated replacement of endogenous segments of varying lengths at the *PPP1R12C* locus with a GFP-based reporter system. (**b**) Efficiency of PREMIER in replacing endogenous segments of different lengths at the *PPP1R12C* locus using an EF1α-GFP reporter system. (**c**) PREMIER-mediated replacement efficiency of endogenous segments of varying sizes at the *PPP1R12C* locus using EF1α-GFP-intron reporter system. (**d**) Schematic illustrating PREMIER-mediated concurrent integration of IRES-GFP at the *GAPDH* locus and EF1α-tdTomato at the *TRAC* locus. (**e**) Efficiency of PREMIER in mediating concurrent integration of IRES-GFP at the *GAPDH* locus and EF1α-tdTomato at the *TRAC* locus. *n* = 3 independent biological replicates. Data are represented as means ± SD. Statistical significance was determined using one-way ANOVA. ns, no significance; *P <.05; **P <.01; ***P <.001; ****P <.0001.

We rigorously evaluated PREMIER’s capacity for replacing endogenous sequences of varying sizes at the *PPP1R12C* locus in HeLa cells using an EF1a-GFP reporter system (Fig. 3A). By incrementally increasing the distance between the pegRNA nick sites, we successfully replaced genomic segments ranging from 56 bp to 10 795 bp. PREMIER demonstrated consistently high replacement efficiency across this wide size spectrum (Fig. 3B and Supplementary Fig. S5A and B), confirming that inter-pegRNA distance on the genome does not significantly limit efficiency and establishing PREMIER’s capability for replacing genomic segments exceeding 10 kb. To further confirm the replacement event at the *PPP1R12C* locus, we subcultured HeLa cells edited by PREMIER, in which a 10 260 bp endogenous sequence was replaced with an EF1a-GFP reporter, and isolated single clones. Of the 19 clones analyzed, 17 exhibited successful amplification of the 5′ and 3′ junction sequences (Supplementary Fig. S5C), and in 3 of the 17 clones, the 10 260 bp endogenous sequence replaced by PREMIER was undetectable (Supplementary Fig. S5D), qPCR verified that the copy number of the insertion cargo in homozygous clones matched that of the replaced sequence in wild-type cells (Supplementary Fig. S5E), indicating homozygous replacement.

To determine the maximum insert size PREMIER can accommodate, we generated EF1a-GFP donor vectors ranging from 2.19 kb to 10.3 kb by inserting progressively larger intronic sequences. PREMIER efficiently mediated the replacement of three distinct genomic segments (112 bp, 1 976 bp, 10 260 bp) at the *PPP1R12C* locus with all donor sizes. Remarkably, even with the largest donor (10.3 kb), PREMIER achieved a high replacement efficiency of 54.7% (Fig. 3C). Finally, we demonstrated PREMIER’s capacity for simultaneous multi-locus editing. Targeting the *GAPDH* locus with an IRES-GFP reporter and the *TRAC* locus with an EF1a-tdTomato reporter (Fig. 3D), we successfully observed cells exhibiting co-integration of both reporters via flow cytometry (Fig. 3E), confirming PREMIER’s ability to facilitate multi-site integration events.

PAINT is a recently developed technology that also leverages PE to achieve large-fragment integration with high efficiency [26], to better reflect the integration efficiency of PREMIER, we compared the efficiency of large-fragment integration mediated by PREMIER, PAINT, and HDR at the *PPP1R12C* and *RPL13A* loci in 293T and HeLa cells. The results showed that PREMIER consistently achieved comparable or higher integration efficiency (Supplementary Fig. S6A). Furthermore, PREMIER-mediated integration was tested in primary porcine embryonic fibroblasts, yielding integration efficiencies of 7.87% at the *ALB* locus and 16.63% at the *GGTA1* locus (Supplementary Fig. S6B), underscore the effectiveness of PREMIER in achieving efficient integration.

### PREMIER exhibits high precision and minimal off-target activity

**Figure 4.**
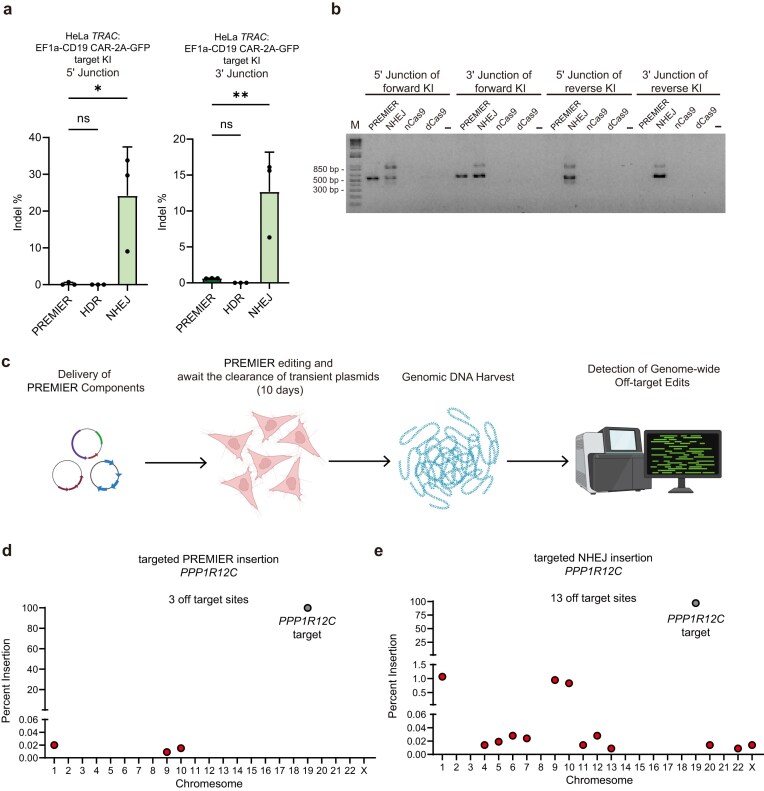
Characterization of precision and off-target in PREMIER-mediated site-specific integration of exogenous sequences. (**a**) Indel frequency at the 5′ junction and 3′ junction of EF1α-CD19 CAR-2A-GFP integrated into the *TRAC* locus in HeLa cells via PREMIER, HDR, and NHEJ. (**b**) PCR-based detection of inversion insertions of IRES-GFP at the *GAPDH* locus mediated by NHEJ and PREMIER. The nCas9 and dCas9 groups served as negative controls, in which nCas9-RT was replaced with nCas9 and dCas9. Primer pairs 5′junction-GAPDH-F1/5′junction-IRES-GFP-Puro-R1 and 3′junction-IRES-GFP-Puro-F1/3′junction-GAPDH-R1 amplify the junctions of forward insertions, whereas primer pairs 5′junction-GAPDH-F1/3′junction-IRES-GFP-Puro-F1 and 5′junction-IRES-GFP-Puro-R1/3′junction-GAPDH-R1 amplify the junctions of reverse insertions. (**c**) Schematic diagram illustrating the genome-wide off-target effects of PREMIER-mediated exogenous sequence integration. (**d**) Average genome-wide integration profile of PREMIER-mediated exogenous sequence integration in mixed cell populations. The target site is located at the *PPP1R12C* locus on chromosome 19. (**e**) Average genome-wide integration profile of NHEJ-mediated exogenous sequence integration in mixed cell populations. The target site is located at the *PPP1R12C* locus on chromosome 19. *n* = 3 independent biological replicates. Data are represented as means ± SD. Statistical significance was determined using one-way ANOVA. ns, no significance; *P <.05; **P <.01; ***P <.001; ****P <.0001.

The precision of PREMIER-mediated integration was assessed at the *TRAC* locus in HeLa cells, comparing junctional indel rates to HDR and NHEJ using next-generation sequencing. PREMIER junctions exhibited indel rates comparable to HDR and significantly lower than NHEJ (Fig. 4A). Analysis of IRES-GFP integration orientation at the *GAPDH* locus via PCR revealed no reverse integration events with PREMIER, in contrast to detectable reverse integration with NHEJ (Fig. 4B)

Genome-wide off-target integration was quantified using Tn5 transposase tagging, junction PCR amplification, and deep sequencing (Fig. 4C). For PREMIER-mediated integration at *PPP1R12C*, the vast majority of sequencing reads (indicating integration events) mapped precisely to the intended target locus (Fig. 4D). In stark contrast, NHEJ-mediated integration showed widespread off-target activity across 13 autosomes, with 3.2% off-target integration rate (Fig. 4E). These results demonstrate PREMIER’s exceptional integration specificity, with off-target rates ∼100-fold lower than NHEJ-based methods.

### PREMIER minimizes genotoxic stress

Conventional HDR and NHEJ rely on DSBs, causing significant DNA damage and impairing proliferation. Utilizing nCas9 (H840A) nickase, PREMIER generates only single-strand nicks, leading us to hypothesize that PREMIER-mediated site-specific integration may cause minimal DNA damage. We assessed the status of DNA DSBs (γ-H2A.X immunofluorescence) and proliferation capacity (Ki67 immunofluorescence) in HeLa cells following integration at the *PPP1R12C* locus. PREMIER-treated cells exhibited γ-H2A.X levels indistinguishable from controls, indicating minimal DNA damage, while HDR and NHEJ groups showed significantly elevated γ-H2A.X signals (Supplementary Fig. S7A and B). Similarly, Ki67 staining revealed unimpaired proliferation in PREMIER and control groups, contrasting with markedly reduced Ki67 intensity in HDR and NHEJ groups (Supplementary Fig. S7C and D).

Previous studies have demonstrated that closely spaced nicks (<100 bp) introduced by nCas9 (H840A) on opposite DNA strands can spontaneously induce unintended DSB formation [41]. To further evaluate whether nick-to-nick distance affects PREMIER-associated DNA damage and cell proliferative potential, we systematically examined both γ-H2A.X and Ki67 immunofluorescence levels across HeLa and HEK293T at PPP1R12C and EEF2 loci. PREMIER configurations were categorized according to inter-nick spacing into three groups: <100 bp, >100 bp, and >200 bp. Our results showed that the PREMIER configuration with a nick distance <100 bp triggered markedly increased γ-H2A.X intensity and reduced Ki67 signals, indicating increased DNA damage and compromised cell proliferation. In contrast, layouts with nick spacing >100 bp and >200 bp, maintained low γ-H2A.X levels and sustained Ki67 intensity comparable to control groups (Supplementary Fig. S7E and F), verifying that appropriate nick separation is essential for PREMIER to avoid DSB induction, minimize genotoxicity, and preserve normal cell proliferation.

### PREMIER-mediated integration involves multiple endogenous repair pathways

In the PREMIER-mediated integration events, multiple interactions may arise between microhomology arms generated by PE and target sites, likely engages multiple DNA repair pathways. Small interfering RNA (siRNA)-mediated knockdown of key repair factors assessed their contribution to PREMIER efficiency at the *GAPDH* locus (IRES-GFP reporter). Knockdown efficacy was confirmed by qPCR (Supplementary Fig. S8A). Depletion of *PARP1* (upstream DSB sensor/recruiter) [42] reduced integration, indicating its role in initiating repair. Knockdown of *POLQ* (DNA polymerase theta, key for MMEJ) [43] and *RAD52* (SSA pathway) [44] caused a moderate decrease, suggesting a supportive but nonessential role for MMEJ. Crucially, knockdown of *XRCC5* (Ku80, essential for NHEJ) [45] significantly reduced efficiency, and knockdown of *RAD51* (central to HDR) [46, 47] nearly abolished integration (Supplementary Fig. S8B). These results demonstrate that PREMIER integration is predominantly mediated by HDR and NHEJ pathways, with a contributory role for MMEJ and SSA, facilitated by the interaction of PE-generated ssDNA microhomology arms with homologous genomic sequences.

### *In vivo* integration and tumor modeling via PREMIER

**Figure 5.**
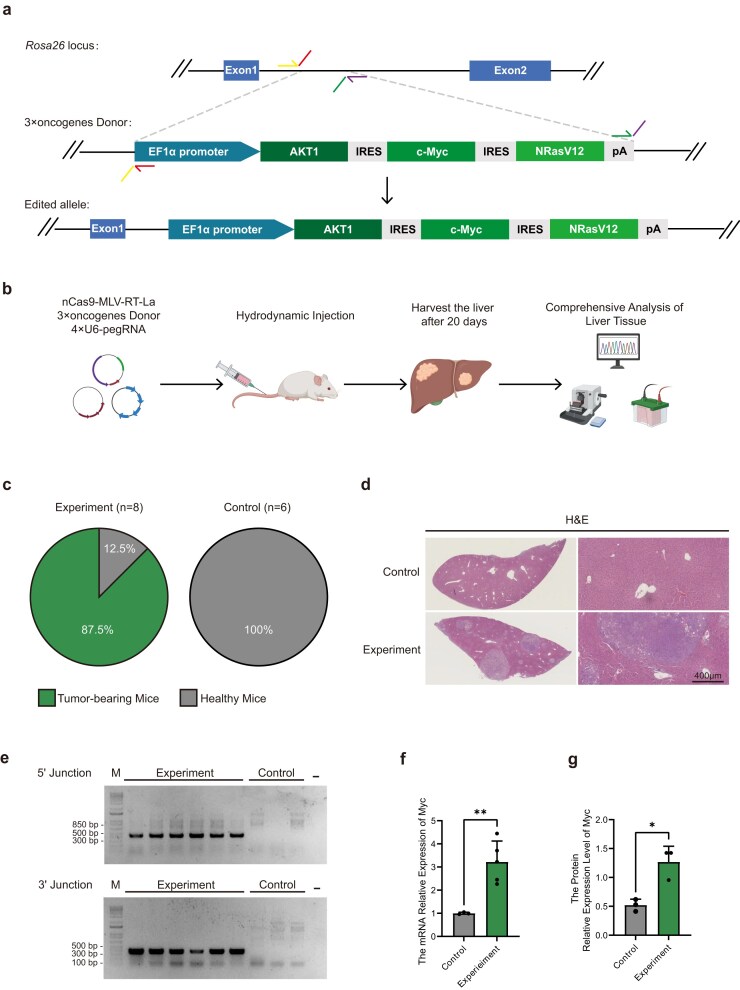
Successful integration of large exogenous sequences into the mouse liver via PREMIER. (**a**) Schematic representation of the integration of an exogenous fragment encoding three oncogenes at the *Rosa26* locus in mouse liver mediated by PREMIER. Yellow, purple, red, and green arrows denote the spacers of pegRNAs targeting the 5′ end of the endogenous target site, the 3′ end of the endogenous target site, the 5′ end of the donor, and the 3′ end of the donor, respectively, along with the direction of transcription indicated. The corresponding microhomology arms transcribed by these pegRNAs are represented by short yellow, purple, red, and green lines. (**b**) Schematic diagram illustrating the hydrodynamic injection of PREMIER components and the 3 × oncogenes donor into mouse liver to induce tumorigenesis and subsequent analysis. (**c**) Pie charts illustrating the proportion of mice exhibiting tumor formation in liver 20 days post-injection in experiment group and control group. Green represents tumor-bearing mice, gray represents healthy mice. (**d**) Histological analysis with H&E staining of mouse liver tumors from the experimental group and mouse liver from the control group. Scale bars, 400 μm. (**e**) PCR genotyping of the junction sites of the *Rosa26* locus in liver tumors from experimental mice and control mouse liver. Primers 5′junction-mRosa26-F/5′junction-EF1a-R2 and 3′junction-SV40pA-F2/3′junction-mRosa26-R amplify the 5′ junction (437 bp) and 3′ junction (361 bp) of the integrated exogenous fragment at the *Rosa26* locus, respectively. (**f**) Comparison of *c-MYC* gene expression levels in tumorous liver tissues from experimental mice and control mice using quantitative reverse transcription polymerase chain reaction (RT-qPCR). The relative mRNA expression levels were calculated by normalizing to *Actb. n* = 5 independent biological replicates. (**g**) Comparison of c-MYC protein expression levels in liver tumors from experimental mice and control mice using western blot. *n* = 3 independent biological replicates. Data are represented as means ± standard error of the mean. The *P*-value was determined using a *t*-test. *P <.05, ***P <.001.

To evaluate PREMIER *in vivo*, we co-delivered PREMIER components (nCas9-RT, four pegRNAs targeting mouse *Rosa26*, and a 6 156 bp donor plasmid encoding oncogenes *AKT1*, c-*MYC*, and *NRAS*^G12V^ in tandem) into ICR mouse livers via hydrodynamic tail vein injection (*n* = 8 experimental, *n* = 6 control; control received nontargeting gRNA). Twenty days post-injection, macroscopic tumors were evident in the livers of 7/8 (87.5%) experimental mice, while no tumors were observed in controls (Fig. 5C and Supplementary Fig. S9A). Histopathological analysis (H&E staining) confirmed tumor development exclusively in the experimental group (Fig. 5D). Genomic DNA extracted from tumors yielded PCR products of expected size for both 5′ and 3′ junctions (Fig. 5E), confirmed by Sanger sequencing (Supplementary Fig. S9B). Furthermore, qRT-PCR and Western blotting revealed significantly elevated c-*MYC* messenger RNA (mRNA) and protein levels in tumor tissue compared to normal liver from control mice (Fig. 5F and G, and Supplementary Fig. S9C), confirming functional expression from the integrated oncogene cassette. Although tumor modeling may not accurately reflect the true efficiency of integration events *in vivo*, it still demonstrates the potential of PREMIER for precise and functional integration of large fragments at an endogenous locus *in vivo*.

### Generation of humanized TP53 chimeric mice via PREMIER

**Figure 6.**
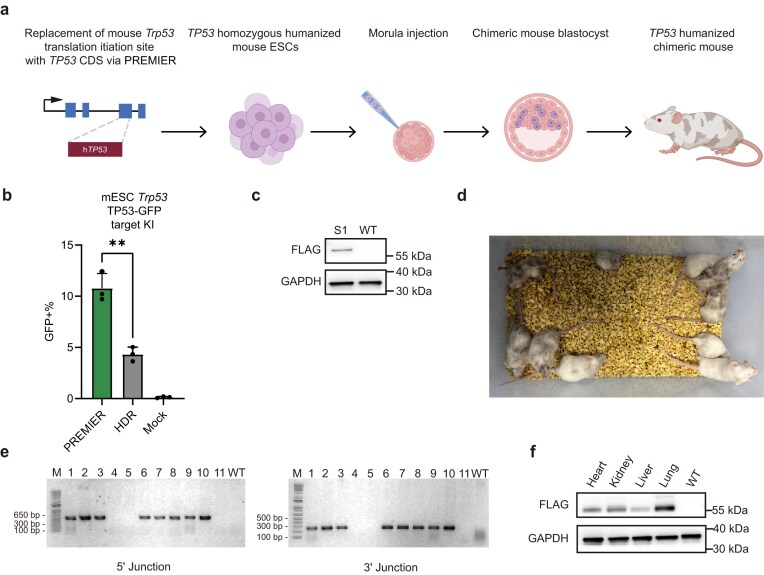
Application of PREMIER in the Construction of Model Animals. (**a**) Schematic depiction of the generation process for humanized *TP53* chimeric mouse models. (**b**) Integration efficiency of a fluorescent reporter system at *Trp53* locus mediated by PREMIER and HDR in mESCs. (**c**) Expression analysis of human p53 protein in homozygous humanized *TP53* embryonic stem (ES) cell lines. (**d**) Image of chimeric mice derived from the chimerization of homozygous humanized *TP53* ES cell lines. (**e**) PCR genotyping of genomic DNA extracted from the tail of humanized *TP53* chimeric mice and wild-type mice. Primers 5′junction-Trp53-F/5′junction-TP53-R and 3′junction-bGHpA-F/-3′junction-Trp53-R amplify the 5′ junction (484 bp) and 3′ junction (253 bp) of the humanized *TP53* sequence at the *Trp53* locus in the chimeric mouse. (**f**) Expression analysis of human p53 protein across various organs in humanized *TP53* chimeric mice.

To showcase PREMIER’s utility in complex genome engineering for model generation, We replaced the translation initiation site-containing exon 3 of murine *Trp53* with the full-length human *TP53* CDS fused to a 3xFLAG tag in mESCs, enabling human p53 expression under the endogenous mouse promoter (Fig. 6A and Supplementary Fig. S10A). We first evaluated its editing efficiency at the murine Trp53 locus in mESCs. We quantified the intrinsic integration efficiency of PREMIER-mediated large-fragment replacement using a fluorescent reporter system. Flow cytometry was used to detect GFP-positive cells, revealing a basal integration efficiency of 10.78% (Fig. 6B).

To further generate precisely edited homozygous mESC clones for constructing humanized animal models efficiently, a loxP-flanked puro selection cassette was placed downstream of the exogenous TP53 CDS to facilitate positive cell enrichment. After transfection of the PREMIER editing components, cells were treated with puromycin to eliminate unedited cells. Surviving cells were then transfected with Cre recombinase to remove the selection cassette. After marker excision, cells were plated at low density for single-colony formation, and 34 independent mESC clones were picked. Genotyping revealed 17 heterozygous (single-copy integration), 13 homozygous (double-copy integration), and 4 negative clones (Supplementary Fig. S10B). A homozygous clone was selected for validation. PCR amplification and Sanger sequencing confirmed precise integration at both junctions, and Western blotting confirmed human p53 protein expression (Fig. 6C).

The well-validated homozygous mESC clone was utilized for morula injection to generate chimeric mice, yielding 11 mice, of which 10 were chimeric (Fig. 6D). Genotyping of genomic DNA from tail confirmed successful integration at both junctions in 8/11 mice (Fig. 6E, and Supplementary Fig. S10C and D). Human p53 protein expression was detected in multiple tissues (heart, liver, spleen, kidney) of a chimeric mouse via Western blotting (Fig. 6F). This successful generation of a humanized *TP53* chimeric mouse model validates PREMIER’s precision and efficiency for large-fragment replacement in complex biological applications.

## Discussion

The precise integration and replacement of large DNA fragment without DSBs has remained a formidable challenge in genome engineering. Here, we present PREMIER, a DSB-free and long-homology-arm-independent CRISPR/Cas-based strategy that harnesses PE to deploy quadruple microhomology arms (30 nt) at genomic and donor termini, enabling efficient (>85% efficiency in cell lines) and precise replacement of sequences exceeding 10 kb. PREMIER outperforms conventional HDR by 10–20-fold across diverse loci and cell types, achieves near-absolute site specificity (100-fold lower off-target integration than NHEJ), and minimizes genotoxic stress—addressing critical limitations of existing editing paradigms.

PREMIER’s design exploits the synergy between programmable nickases and RT activity to generate complementary single-stranded microhomology arms. Optimization of pegRNA architecture (e.g. RTT length, La-MLV-RT fusion) and donor targeting enhanced efficiency by stabilizing editing intermediates.Notably, our modular optimization from PREMIER v0 to v3 revealed that each individual modification—incorporation of the La domain into MLV-RT (v1) and adoption of extended pegRNA (epegRNA, v2)—independently yielded prominent improvement in integration efficiency. Nevertheless, combining both modifications in PREMIER v3 did not produce further synergistic gains, with editing efficiency remaining comparable to v1 and v2. We speculate that both the La domain and epegRNA improve editing efficiency by enhancing pegRNA stability, and their functional mechanisms are largely overlapping. Consistent with our observation, previous studies on La-assisted PE also demonstrated that combining the La domain with epegRNA fails to further elevate editing efficiency [38], as the two elements act through the same regulatory pathway to stabilize pegRNA, leading to efficiency saturation when applied in combination.

Crucially, PREMIER operates independently of DSB repair pathways yet engages multiple endogenous mechanisms: *RAD51*-dependent HDR and *XRCC5*-mediated NHEJ dominate integration, with *POLQ*-driven MMEJ playing a subsidiary role. This multipathway engagement ensures robustness while avoiding the chromosomal aberrations and p53 activation typical of DSB-dependent editors.

PREMIER’s capacity to replace genomic segments from 56 bp to 10.8 kb without efficiency loss overcomes a key size constraint in precision editing. Its ability to orchestrate simultaneous multilocus integrations (e.g. dual fluorescent reporters at *GAPDH* and *TRAC*) underscores modularity for complex engineering. In vivo, PREMIER mediated hepatocyte-specific tumorigenesis via seamless *Rosa26*-directed integration of a 6.2-kb triple-oncogene cassette (*AKT1*/c-*MYC*/*NRAS*^G12V^) with 87.5% penetrance, demonstrating utility in somatic genome manipulation.

Beyond its technical merits, PREMIER enables biological models. By replacing murine *Trp53* exon 3 with full-length human *TP53* CDS in ES cells, we established the first PE-derived humanized p53 chimeric mice—validating PREMIER’s capacity for cross-species gene replacement. This approach sidesteps embryonic lethality risks associated with conventional transgenics and offers a blueprint for humanizing other pleiotropic genes.

While this manuscript was under review, a recent study reported a QuadPE strategy that similarly employs four pegRNAs to generate 3′ flaps at both the genomic target and donor DNA ends, enabling efficient and precise large-fragment genomic integration [48]. In the QuadPE system, the genomic flap is designed to be complementary to the donor flap. In contrast, our PREMIER system utilizes a distinct homology-based flap design, in which the genomic flap is homologous to the sequence upstream of the nick site on the donor, and the donor flap is homologous to the sequence upstream of the nick site on the genome, without direct complementarity between the two flaps. We demonstrate that our alternative flap configuration also supports comparably high-efficiency and precise site-specific integration.

A key advantage of the PREMIER design is its ability to achieve seamless integration: by engineering the pegRNA target sequences at both ends of the donor to closely resemble the corresponding genomic sequences (with only minimal point mutations introduced to prevent cross-targeting between pegRNAs), no extraneous complementary sequences are introduced at the insertion junctions. Furthermore, we show that PREMIER enables efficient replacement of endogenous genomic sequences at the locus level (>10 kb) and exhibits potential for *in vivo* editing and the generation of animal models.

Nevertheless, limitations of our current validation should be noted, the *in vivo* tumor formation assay also has inherent limitations for evaluating editing efficiency. We observed a high tumor incidence (7/8 mice) following PREMIER-mediated integration of the oncogene cassette at the Rosa26 locus,but this metric should not be interpreted as a direct quantitative measure of *in vivo* editing efficiency. Tumor formation can be driven by successful editing in only a very small fraction of hepatocytes, followed by strong positive selection due to the potent oncogenic program. Therefore, the tumor incidence likely overestimates the actual editing rate in the liver.

While PREMIER demonstrates enhanced efficiency and specificity compared to classical methods, delivery optimization—particularly for *in vivo* applications—warrants further study. Miniaturizing components (e.g. compact Cas9-RT fusions) could enhance viral packaging efficacy. Additionally, elucidating cell-type-specific repair biases may refine PREMIER’s design rules. Our results revealed that RAD51 significantly impacts the integration efficiency mediated by PREMIER. Given that RAD51 expression is often suppressed in many cell types *in vivo*, this may hinder PREMIER’s integration efficiency in such settings. Thus, enhancing PREMIER’s performance in *in vivo* applications is an urgent issue to address.

Small molecules that regulate the cell cycle or inhibit specific repair pathway proteins have been shown to boost editing efficiency *in vitro* [10, 20]. However, their lack of specificity limits their applicability in *in vivo* editing. A recent study on PE demonstrated increased efficiency by fusing a small molecule protein that inhibits the mismatch repair pathway [49]. This approach provides a potential strategy for improving PREMIER’s efficiency *in vivo* by incorporating small molecule proteins that inhibit specific repair pathways, thereby addressing the challenge of low editing efficiency in *in vivo* contexts.

## Supplementary Material

gkag626_Supplemental_Files

## Data Availability

The data underlying this article are available in the article and in its online supplementary material. The sequencing data underlying this article are available in the National Center for Biotechnology Information (NCBI) Sequence Read Archive (SRA), and can be accessed with project accession numbers PRJNA1370196.
