# Supplementary material for: Prime editing-mediated microhomology enables efficient replacement of large DNA
Source: Nucleic Acids Res. 2026 Jun 22;54(12):gkag626. doi: 10.1093/nar/gkag626 (PMC13284714; doi:10.1093/nar/gkag626)
Supplement: gkag626_Supplemental_Files [file gkag626_supplemental_files.zip › supplymentary figures S1-10(1).pdf]

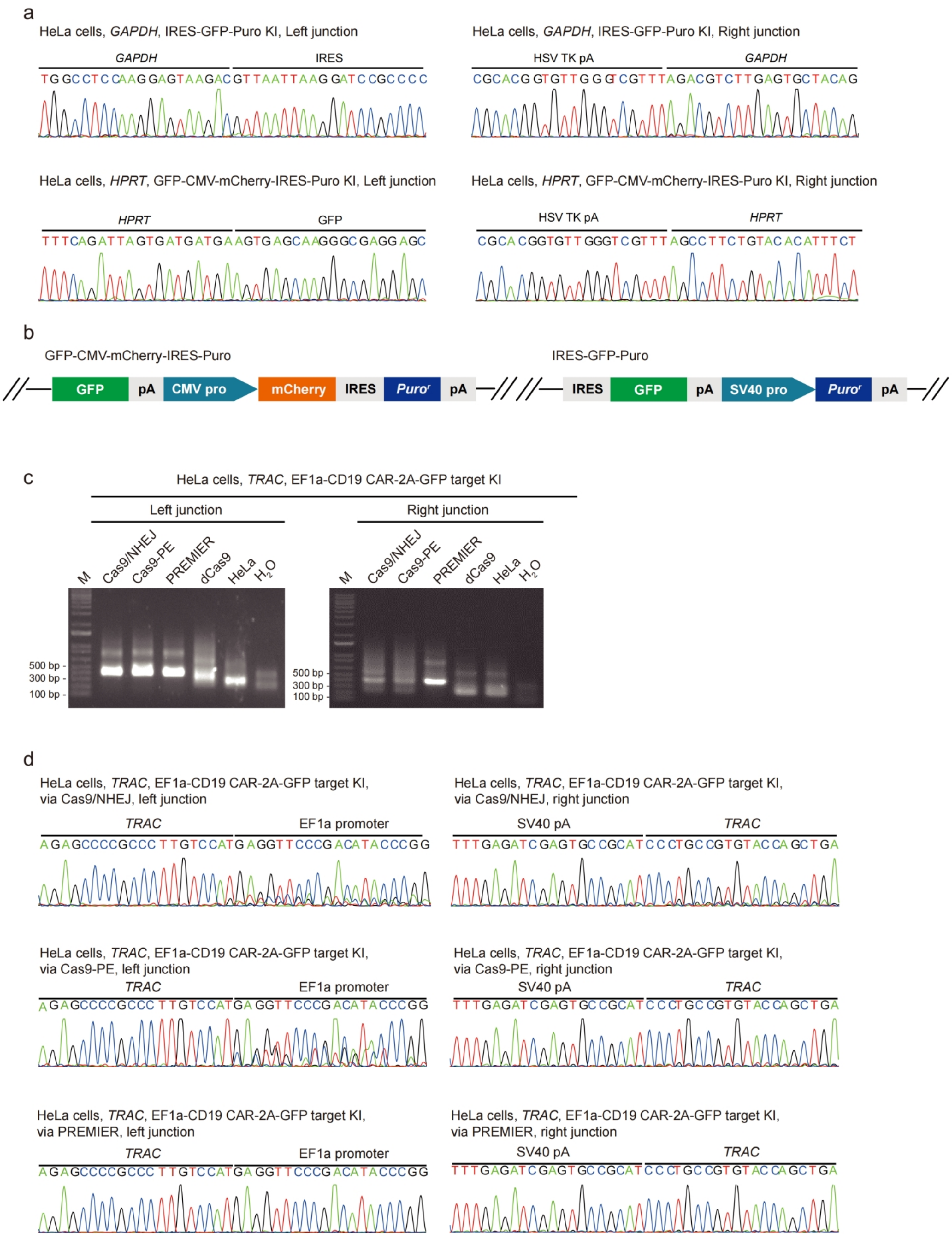
a
HeLa cells, GAPDH, IRES-GFP-Puro KI, Left junction
GAPDH
IRES
TGGCCTCCAAGGAGTAAGACGTTAATTAAGGATCCGCCCC
HeLa cells, GAPDH, IRES-GFP-Puro KI, Right junction
HSV TK pA
GAPDH
CGCACGGTGTTGGGTCGTTTAGACGTCTTGAGTGCTACAG
HeLa cells, HPRT, GFP-CMV-mCherry-IRES-Puro KI, Left junction
HPRT
GFP
TTTCAGATTAGTGATGATGAAGTGAGCAAGGGCGAGGAGC
HeLa cells, HPRT, GFP-CMV-mCherry-IRES-Puro KI, Right junction
HSV TK pA
HPRT
CGCACGGTGTTGGGTCGTTTAGCCTTCTGTACACATTTCT
b
GFP-CMV-mCherry-IRES-Puro
GFP
pA
CMV pro
mCherry
IRES
Puro^r
pA
IRES-GFP-Puro
IRES
GFP
pA
SV40 pro
Puro^r
pA
c
HeLa cells, TRAC, EF1a-CD19 CAR-2A-GFP target KI
Left junction
Right junction
M
Cas9/NHEJ
Cas9-PE
PREMIER
dCas9
HeLa
H2O
500 bp
300 bp
100 bp
d
HeLa cells, TRAC, EF1a-CD19 CAR-2A-GFP target KI, via Cas9/NHEJ, left junction
TRAC
EF1a promoter
AGAGCCCCGCCCTTGTCCATGAGGTTCCCGACATACCCGG
HeLa cells, TRAC, EF1a-CD19 CAR-2A-GFP target KI, via Cas9/NHEJ, right junction
SV40 pA
TRAC
TTTGAGATCGAGTGCCGCATCCCTGCCGTGTACCAGCTGA
HeLa cells, TRAC, EF1a-CD19 CAR-2A-GFP target KI, via Cas9-PE, left junction
TRAC
EF1a promoter
AGAGCCCCGCCCTTGTCCATGAGGTTCCCGACATACCCGG
HeLa cells, TRAC, EF1a-CD19 CAR-2A-GFP target KI, via Cas9-PE, right junction
SV40 pA
TRAC
TTTGAGATCGAGTGCCGCATCCCTGCCGTGTACCAGCTGA
HeLa cells, TRAC, EF1a-CD19 CAR-2A-GFP target KI, via PREMIER, left junction
TRAC
EF1a promoter
AGAGCCCCGCCCTTGTCCATGAGGTTCCCGACATACCCGG
HeLa cells, TRAC, EF1a-CD19 CAR-2A-GFP target KI, via PREMIER, right junction
SV40 pA
TRAC
TTTGAGATCGAGTGCCGCATCCCTGCCGTGTACCAGCTGA

Supplementary Figure. S1. Gel electrophoresis and Sanger sequencing verification of the knock-in-specific PCR products, related to Figure 1.

(a) Sanger sequencing chromatograms of genomic junctions from the V4 knock-in experiment at *HPRT* and *GAPDH* sites. (b) The schematic diagrams of the two reporter cassettes employed in this study to evaluate the knock-in efficiency of various strategies. (c) Agarose gel results of knock-in-specific junction PCR at the *TRAC* locus using the Cas9/NHEJ, Cas9-PE, and PREMIER. (d) The Sanger sequencing chromatograms of genomic junctions at the *TRAC* locus from the Cas9/NHEJ, Cas9-PE, and PREMIER approaches.

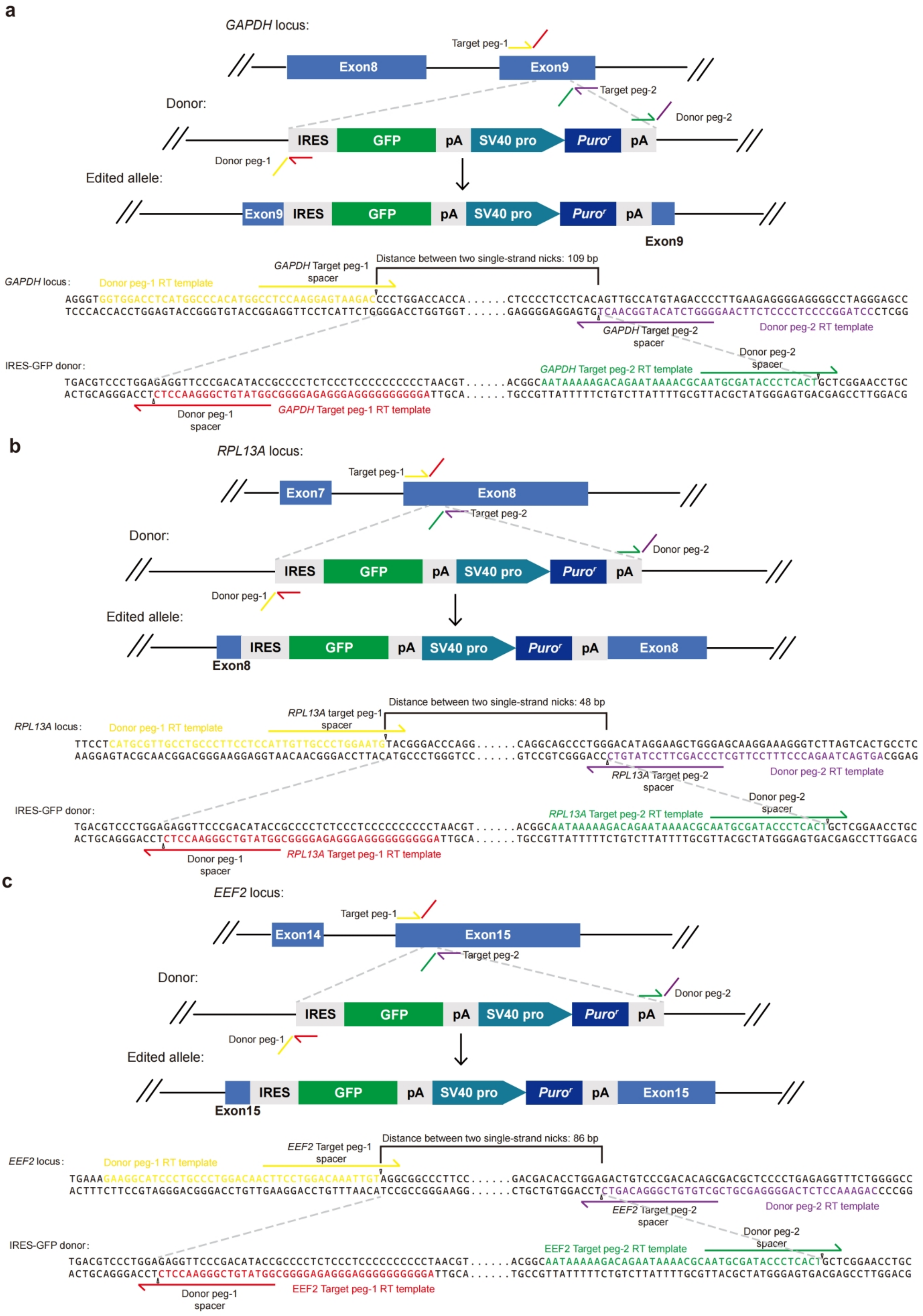
a
GAPDH locus:
Target peg-1
Exon8
Exon9
Target peg-2
Donor:
Donor peg-2
IRES
GFP
pA
SV40 pro
Puro^r
pA
Donor peg-1
Edited allele:
Exon9
IRES
GFP
pA
SV40 pro
Puro^r
pA
Exon9
Distance between two single-strand nicks: 109 bp
GAPDH Target peg-1 spacer
GAPDH locus:
Donor peg-1 RT template
AGGGTGGTGGACCTCATGGCCCACATGGCCTCCAAGGAGTAAGACCCCTGGACCACCA......CTCCCCTCCTCACAGTTGCCATGTAGACCCCTTGAAGAGGGGAGGGGCCTAGGGAGCC
TCCCACCACCTGGAGTACCGGGTGTACCGGAGGTTCCTCATTCTGGGGACCTGGTGGT......GAGGGGAGGAGTGTCAACGGTACATCTGGGGAACTTCTCCCCTCCCCGGATCCCTCGG
GAPDH Target peg-2 spacer
Donor peg-2 RT template
Donor peg-2 spacer
IRES-GFP donor:
GAPDH Target peg-2 RT template
TGACGTCCCTGGAGAGGTTCCCGACATACCGCCCCTCTCCCTCCCCCCCCCCTAACGT......ACGGCAATAAAAAGACAGAATAAAACGCAATGCGATACCCTCACTGCTCGGAACCTGC
ACTGCAGGGACCTCTCCAAGGGCTGTATGGCGGGGAGAGGGAGGGGGGGGGGATTGCA......TGCCGTTATTTTTCTGTCTTATTTTGCGTTACGCTATGGGAGTGACGAGCCTTGGACG
Donor peg-1 spacer
GAPDH Target peg-1 RT template
b
RPL13A locus:
Target peg-1
Exon7
Exon8
Target peg-2
Donor:
Donor peg-2
IRES
GFP
pA
SV40 pro
Puro^r
pA
Donor peg-1
Edited allele:
IRES
GFP
pA
SV40 pro
Puro^r
pA
Exon8
Exon8
Distance between two single-strand nicks: 48 bp
RPL13A target peg-1 spacer
RPL13A locus:
Donor peg-1 RT template
TTCCTCATGCGTTGCCTGCCCTTCCTCCATTGTTGCCCTGGAATGTACGGGACCCAGG......CAGGCAGCCCTGGGACATAGGAAGCTGGGAGCAAGGAAAGGGTCTTAGTCACTGCCTC
AAGGAGTACGCAACGGACGGGAAGGAGGTAACAACGGGACCTTACATGCCCTGGGTCC......GTCCGTCGGGACCCTGTATCCTTCGACCCTCGTTCCTTTCCCAGAATCAGTGACGGAG
RPL13A Target peg-2 spacer
Donor peg-2 RT template
Donor peg-2 spacer
IRES-GFP donor:
RPL13A Target peg-2 RT template
TGACGTCCCTGGAGAGGTTCCCGACATACCGCCCCTCTCCCTCCCCCCCCCCTAACGT......ACGGCAATAAAAAGACAGAATAAAACGCAATGCGATACCCTCACTGCTCGGAACCTGC
ACTGCAGGGACCTCTCCAAGGGCTGTATGGCGGGGAGAGGGAGGGGGGGGGGATTGCA......TGCCGTTATTTTTCTGTCTTATTTTGCGTTACGCTATGGGAGTGACGAGCCTTGGACG
Donor peg-1 spacer
RPL13A Target peg-1 RT template
c
EEF2 locus:
Target peg-1
Exon14
Exon15
Target peg-2
Donor:
Donor peg-2
IRES
GFP
pA
SV40 pro
Puro^r
pA
Donor peg-1
Edited allele:
IRES
GFP
pA
SV40 pro
Puro^r
pA
Exon15
Exon15
Distance between two single-strand nicks: 86 bp
EEF2 Target peg-1 spacer
EEF2 locus:
Donor peg-1 RT template
TGAAAGAAGGCATCCCTGCCCTGGACAACTTCCTGGACAAATTGTAGGCGGCCCTTCC......GACGACACCTGGAGACTGTCCCGACACAGCGACGCTCCCCTGAGAGGTTTCTGGGGCC
ACTTTCTTCCGTAGGGACGGGACCTGTTGAAGGACCTGTTTAACATCCGCCGGGAAGG......CTGCTGTGGACCTCTGACAGGGCTGTGTCGCTGCGAGGGGACTCTCCAAAGACCCCGG
EEF2 Target peg-2 spacer
Donor peg-2 RT template
Donor peg-2 spacer
IRES-GFP donor:
EEF2 Target peg-2 RT template
TGACGTCCCTGGAGAGGTTCCCGACATACCGCCCCTCTCCCTCCCCCCCCCCTAACGT......ACGGCAATAAAAAGACAGAATAAAACGCAATGCGATACCCTCACTGCTCGGAACCTGC
ACTGCAGGGACCTCTCCAAGGGCTGTATGGCGGGGAGAGGGAGGGGGGGGGGATTGCA......TGCCGTTATTTTTCTGTCTTATTTTGCGTTACGCTATGGGAGTGACGAGCCTTGGACG
Donor peg-1 spacer
EEF2 Target peg-1 RT template

Supplementary Figure. S2. Schematic representation of integration mediated by PREMIER at various loci in Hela cells.

(a) Schematic diagram showing the integration of the IRES-GFP cassette into the 3' UTR of *GAPDH* under the mediation of PREMIER. The diagram illustrates the four pegRNA spacer regions targeting the integration site and the donor sequence, as well as the homologous sequences of the microhomology arms generated by reverse transcription. The positions of nicks introduced by the pegRNAs are indicated by triangles. (b) Schematic diagram showing the integration of the IRES-GFP cassette into the 3' UTR of *RPL13A* under the mediation of PREMIER. The diagram illustrates the four pegRNA spacer regions targeting the integration site and the donor sequence, as well as the homologous sequences of the microhomology arms generated by reverse transcription. The positions of nicks introduced by the pegRNAs are indicated by triangles. (c) Schematic diagram showing the integration of the IRES-GFP cassette into the 3' UTR of *EEF2* under the mediation of PREMIER. The diagram illustrates the four pegRNA spacer regions targeting the integration site and the donor sequence, as well as the homologous sequences of the microhomology arms generated by reverse transcription. The positions of nicks introduced by the pegRNAs are indicated by triangles.

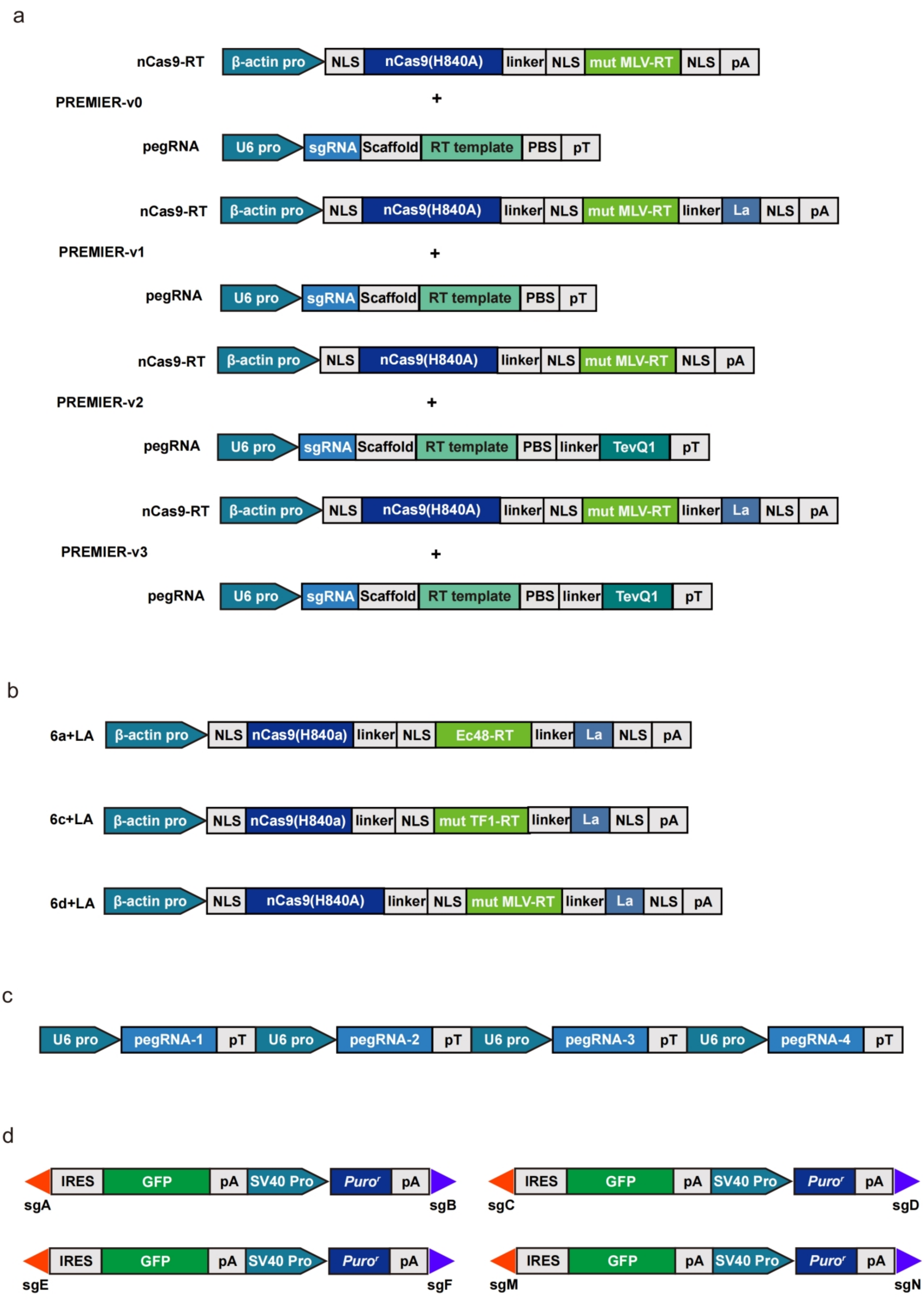
a
nCas9-RT
β-actin pro
NLS
nCas9(H840A)
linker
NLS
mut MLV-RT
NLS
pA
PREMIER-v0
+
pegRNA
U6 pro
sgRNA
Scaffold
RT template
PBS
pT
nCas9-RT
β-actin pro
NLS
nCas9(H840A)
linker
NLS
mut MLV-RT
linker
La
NLS
pA
PREMIER-v1
+
pegRNA
U6 pro
sgRNA
Scaffold
RT template
PBS
pT
nCas9-RT
β-actin pro
NLS
nCas9(H840A)
linker
NLS
mut MLV-RT
NLS
pA
PREMIER-v2
+
pegRNA
U6 pro
sgRNA
Scaffold
RT template
PBS
linker
TevQ1
pT
nCas9-RT
β-actin pro
NLS
nCas9(H840A)
linker
NLS
mut MLV-RT
linker
La
NLS
pA
PREMIER-v3
+
pegRNA
U6 pro
sgRNA
Scaffold
RT template
PBS
linker
TevQ1
pT
b
6a+LA
β-actin pro
NLS
nCas9(H840a)
linker
NLS
Ec48-RT
linker
La
NLS
pA
6c+LA
β-actin pro
NLS
nCas9(H840a)
linker
NLS
mut TF1-RT
linker
La
NLS
pA
6d+LA
β-actin pro
NLS
nCas9(H840A)
linker
NLS
mut MLV-RT
linker
La
NLS
pA
c
U6 pro
pegRNA-1
pT
U6 pro
pegRNA-2
pT
U6 pro
pegRNA-3
pT
U6 pro
pegRNA-4
pT
d
IRES
GFP
pA
SV40 Pro
Puro^r
pA
sgA
sgB
IRES
GFP
pA
SV40 Pro
Puro^r
pA
sgC
sgD
IRES
GFP
pA
SV40 Pro
Puro^r
pA
sgE
sgF
IRES
GFP
pA
SV40 Pro
Puro^r
pA
sgM
sgN

Supplementary Figure. S3. Optimization strategies for PREMIER, related to Figure 2.

(a) Schematic diagram of the design and optimization of PREMIER. (b) Schematic diagram of plasmid constructs where the reverse transcriptase (RT) component of the nCas9-RT in PREMIER has been replaced with various truncated RT variants of PE6. (c) Schematic diagram of the construction of a single plasmid designed to co-express four pegRNAs. (d) Schematic diagram of the construction of the donor with different pegRNA target sites (sgAB, sgCD, sgEF, sgMN).

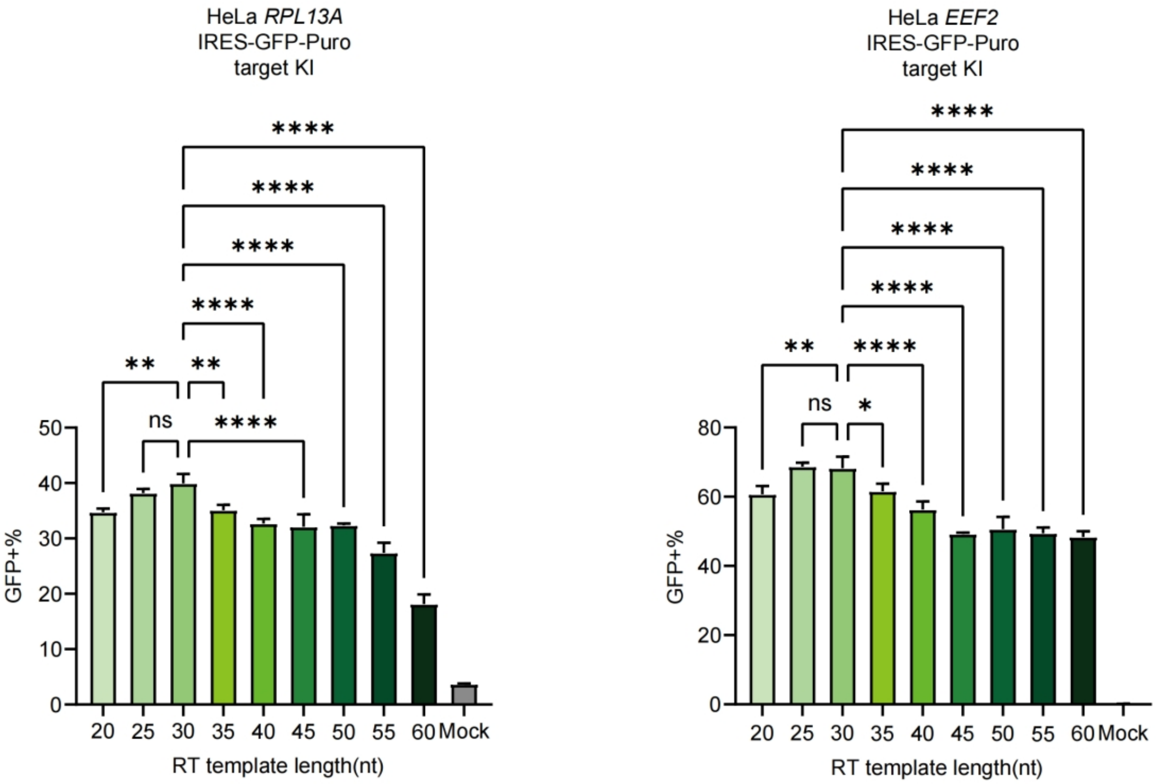

Supplementary Figure. S4. Assessment of the impact of microhomology arm length on integration efficiency mediated by PREMIER, related to Figure 2

Integration efficiency of IRES-GFP at the *RPL13A* and *EEF2* loci in HeLa cells by PREMIER with varying lengths of the reverse transcription template in pegRNAs. n = 3 independent biological replicates. Data are represented as means ± SD. Statistical significance was determined using one-way ANOVA. ns, no significance, *p < 0.05, **p < 0.01, ****p < 0.0001.

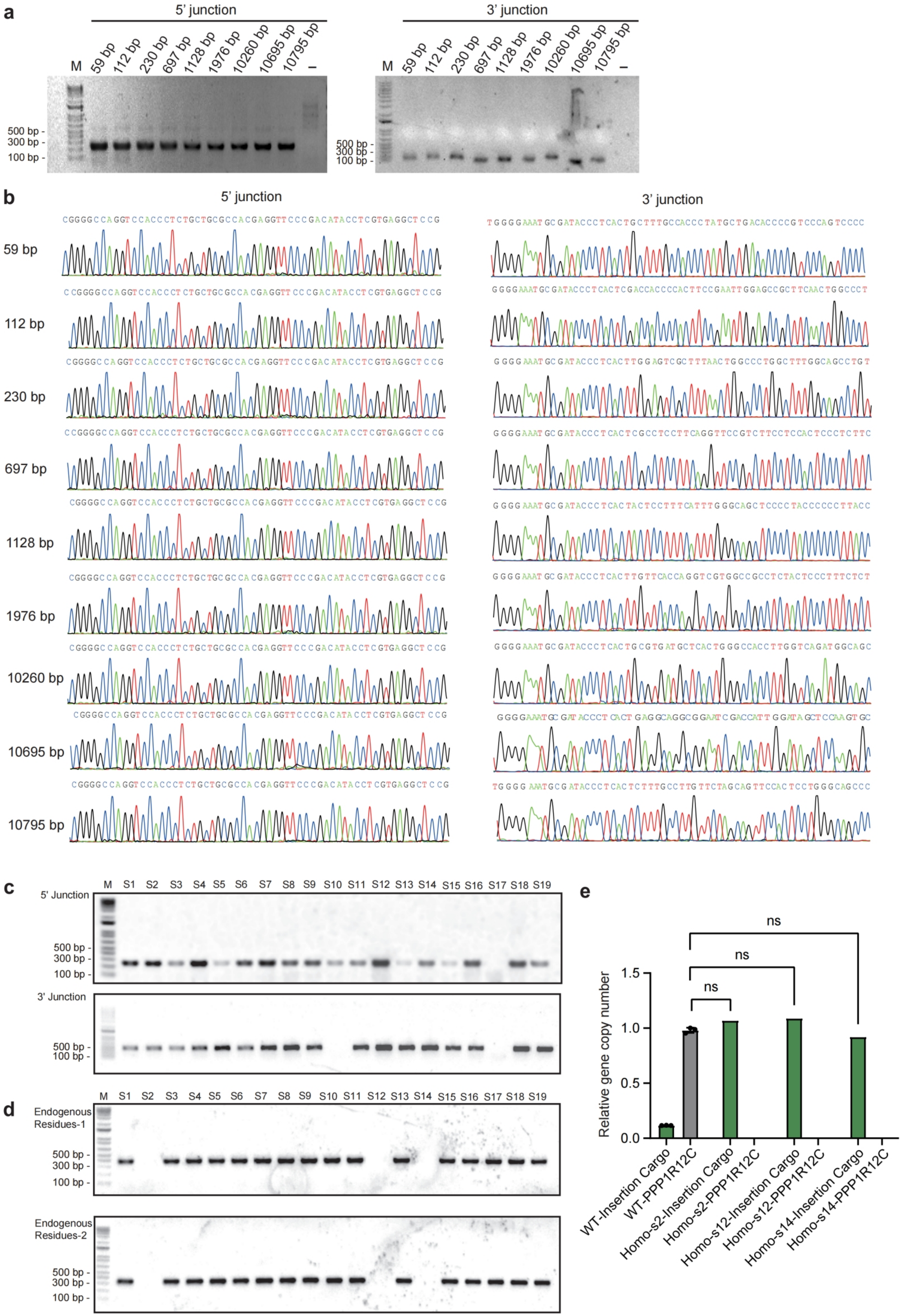

a
5' junction
3' junction
M
59 bp
112 bp
230 bp
697 bp
1128 bp
1976 bp
10260 bp
10695 bp
10795 bp
–
500 bp -
300 bp -
100 bp -
b
5' junction
3' junction
59 bp
112 bp
230 bp
697 bp
1128 bp
1976 bp
10260 bp
10695 bp
10795 bp
c
5' Junction
3' Junction
M S1 S2 S3 S4 S5 S6 S7 S8 S9 S10 S11 S12 S13 S14 S15 S16 S17 S18 S19
500 bp -
300 bp -
100 bp -
d
Endogenous Residues-1
Endogenous Residues-2
e
Relative gene copy number
0.0
0.5
1.0
1.5
ns
ns
ns
WT-Insertion Cargo
WT-PPP1R12C
Homo-s2-Insertion Cargo
Homo-s2-PPP1R12C
Homo-s12-Insertion Cargo
Homo-s12-PPP1R12C
Homo-s14-Insertion Cargo
Homo-s14-PPP1R12C

Supplementary Figure. S5. Genotyping results at junctions following PREMIER-mediated replacement of endogenous sequences with various sizes

(a) PCR-based genotyping of the 5' and 3' junctions after PREMIER-mediated replacement of endogenous sequences of various sizes with exogenous sequences. (b) Sanger sequencing results at the 5' and 3' junctions after PREMIER-mediated replacement of endogenous sequences of various sizes with exogenous sequences. (c) PCR-based genotyping of the 5' and 3' junctions of HeLa cells edited at the *PPP1R12C* locus by PREMIER, with a 10260 bp endogenous sequence replaced by EF1a-GFP reporter. The primers 5'junction-hPPP1R12C-F/5'junction-EF1a-R2 and 3'junction-bGHpA-F/3'junction-nick-distance-10260bp-hPPP1R12C-R amplify the 5' junction (239 bp) and 3' junction (443 bp) between EF1a-GFP reporter and the endogenous sequence at the *PPP1R12C*, respectively. (d) PCR-based genotyping of the *PPP1R12C* locus sequence of HeLa cells that replaced by PREMIER. The primers WT-1-hPPP1R12C-F/ WT-1-hPPP1R12C-R and WT-2-hPPP1R12C-F/WT-2-hPPP1R12C-R amplify the 5' junction (377 bp) and 3' junction (342 bp). (e) qPCR-based comparison of relative gene copy number at the PPP1R12C locus in wild-type (WT) cells and homozygous knock-in clones. Relative PPP1R12C copy number in wild-type cells and relative insertion cargo copy number in homozygous knock-in clones (Homo-s2, s12, and s14) were quantified by qPCR. All values were normalized to the WT-PPP1R12C group, which was set to 1. n = 3 independent biological replicates for WT groups, with three technical replicates per biological replicate. Statistical significance was assessed using Student’s t-test. ns, not significant.

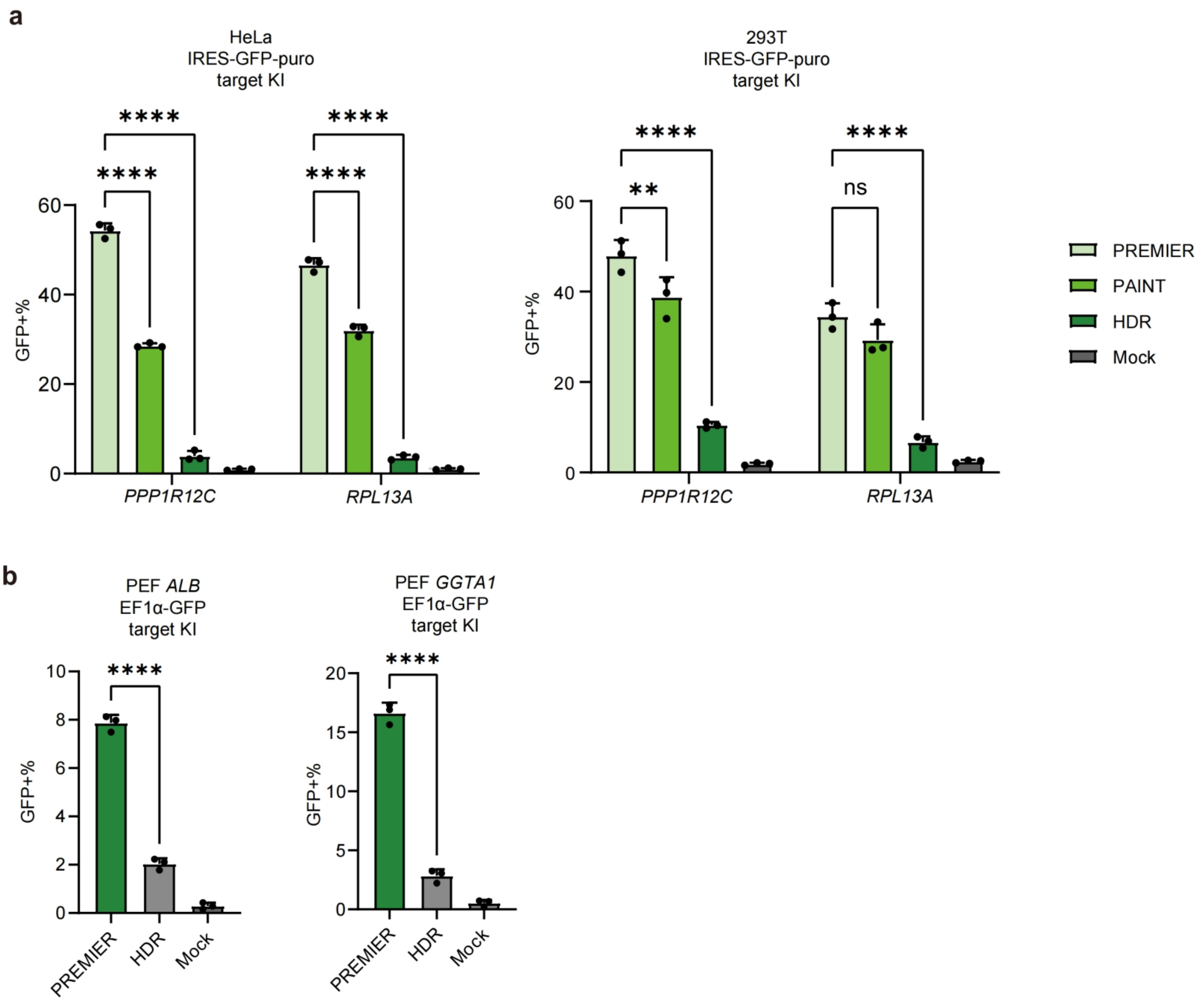

Supplementary Figure. S6. Comparison of fluorescent reporter integration efficiency across different cell types and loci using multiple editing strategies.

(a) Integration efficiency of a fluorescent reporter system at RPL13A and EEF2 loci mediated by PREMIER, PAINT and NHEJ in 293T and HeLa cell types. (b) Integration efficiency of a fluorescent reporter system at the ALB and GGTA1 loci in porcine embryonic fibroblasts (PEF), mediated by PREMIER and HDR strategies.

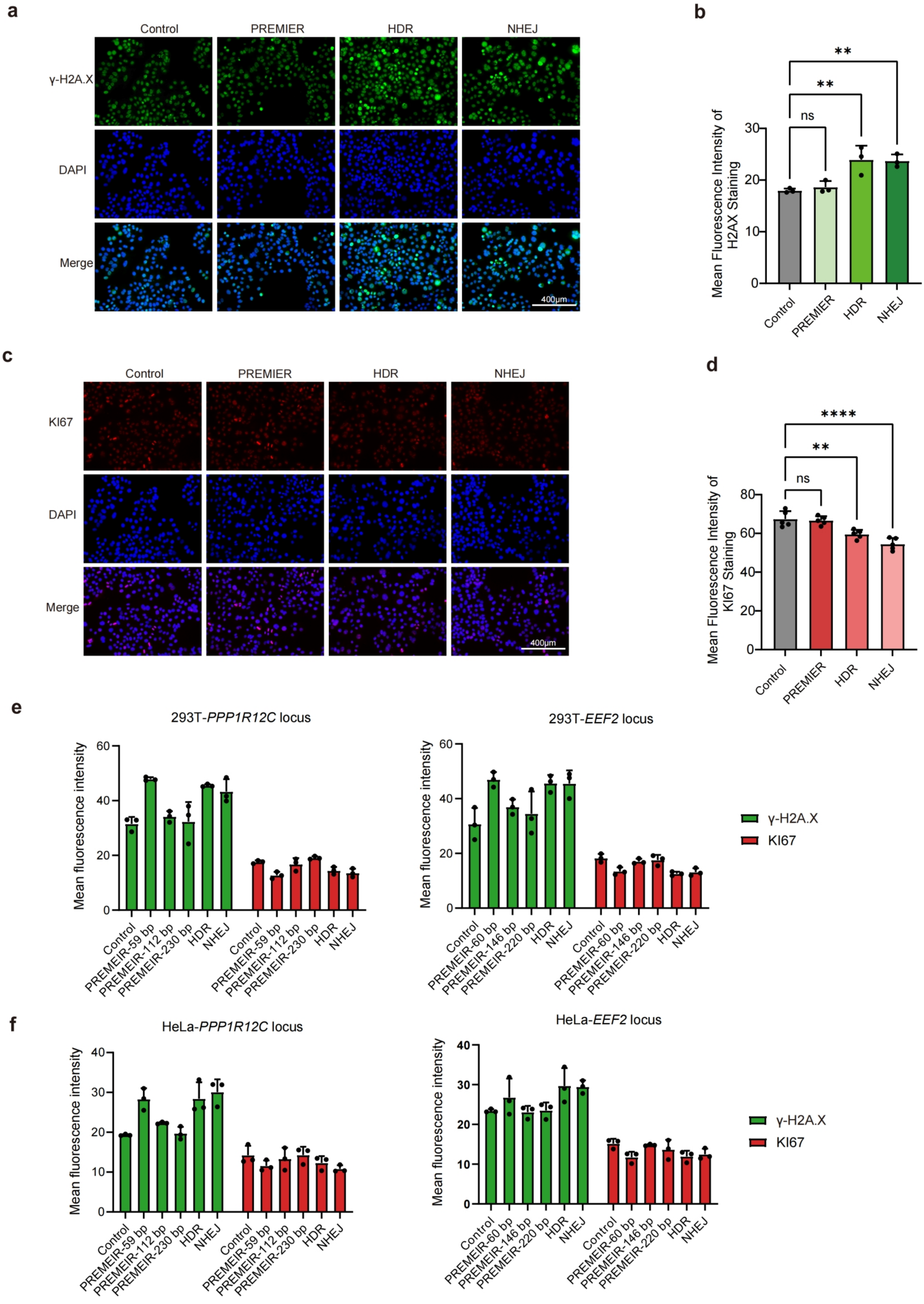

a
Control
PREMIER
HDR
NHEJ
γ-H2A.X
DAPI
Merge
400μm
b
Mean Fluorescence Intensity of H2AX Staining
ns
**
**
c
KI67
d
Mean Fluorescence Intensity of KI67 Staining
****
e
293T-PPP1R12C locus
293T-EEF2 locus
Mean fluorescence intensity
PREMEIR-59 bp
PREMEIR-112 bp
PREMEIR-230 bp
PREMEIR-60 bp
PREMEIR-146 bp
PREMEIR-220 bp
γ-H2A.X
KI67
f
HeLa-PPP1R12C locus
HeLa-EEF2 locus

Supplementary Figure. S7. Assessment of gene-editing-induced DNA damage and impact on cell proliferation via immunofluorescence.

(a) Immunofluorescence staining of γ-H2A.X to indicate the level of DNA damage in HeLa cells transfected with components targeting the *PPP1R12C* locus, including PREMIER, HDR, NHEJ, and control groups. Nuclei are stained with DAPI. Scale bars, 400 μm. (b) Quantification of DNA damage caused by PREMIER, HDR, and NHEJ methods following exogenous sequence integration in HeLa cells, as measured by immunofluorescence staining for γ-H2A.X. Fluorescence intensity was calculated based on γ-H2A.X fluorescence intensity per DAPI-stained area. n = 3 technical replicates. (c) Immunofluorescence staining of KI67 to indicate cell proliferation capacity in HeLa cells transfected with components targeting the *PPP1R12C* locus, including PREMIER, HDR, NHEJ, and control groups. Nuclei are stained with DAPI. Scale bars, 400 μm. (d) Quantification of cell proliferation following integration of exogenous sequences mediated by PREMIER, HDR, and NHEJ in HeLa cells, as measured by Ki67 immunofluorescence staining. Fluorescence intensity was calculated based on KI67 fluorescence intensity per DAPI-stained area. n = 5 technical replicates. (e)Quantification of DNA damage and cell proliferation induced by PREMIER strategies with different nick-to-nick distances, alongside HDR and NHEJ controls, at the PPP1R12C and EEF2 loci in HEK293T cells. n = 3 technical replicates. (f) Quantification of DNA damage and cell proliferation induced by PREMIER strategies with different nick-to-nick distances, alongside HDR and NHEJ controls, at the PPP1R12C and EEF2 loci in HeLa cells. n = 3 technical replicates. Data are represented as means ± SD. The p-value was determined using one-way ANOVA. ns no significance, ** $p<0.01$ and **** $p<0.0001$.

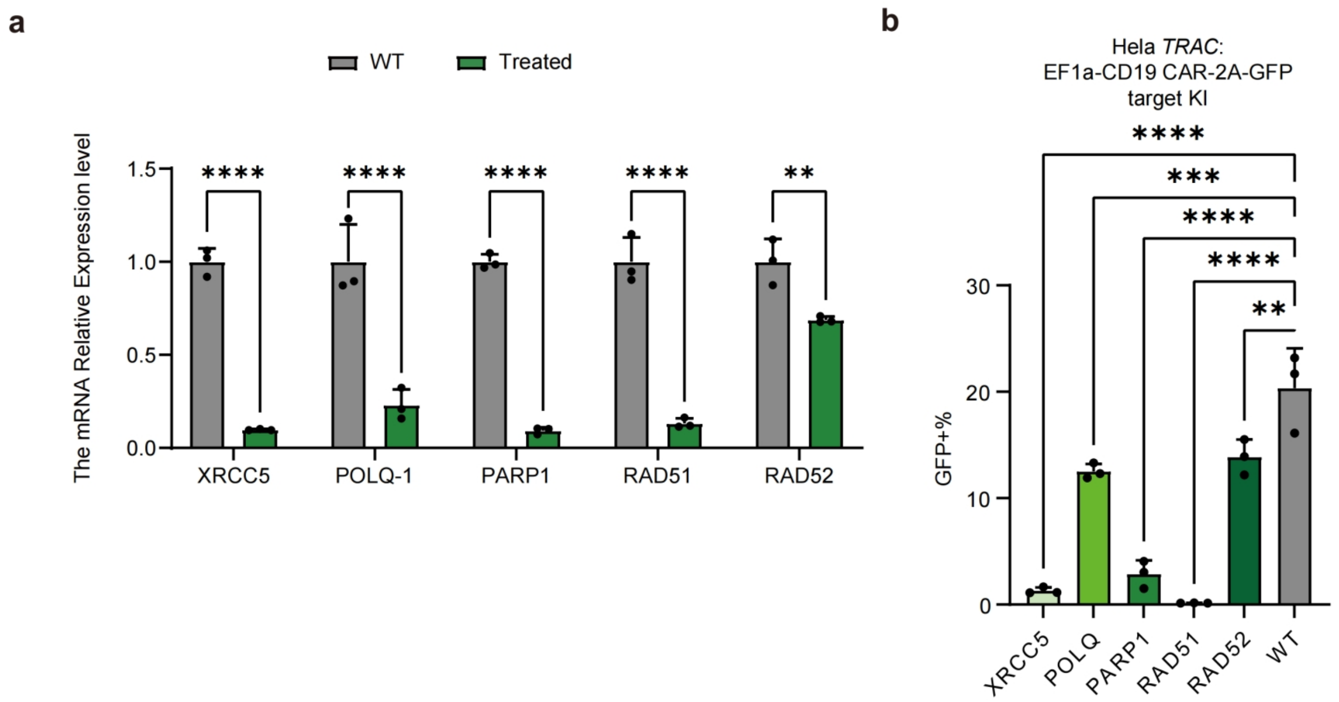

Supplementary Figure. S8. Mechanisms of PREMIER-mediated Exogenous Sequence integration

(a) qPCR analysis of shRNA-mediated knockdown efficiency of DNA repair genes in HeLa cells. Knockdown of *XRCC5*, *POLQ*, *PARP1*, *RAD51*, and *RAD52* was confirmed by significant reduction in their mRNA levels in treated cells compared to wild-type controls. The relative mRNA expression levels were calculated by normalizing to *GAPDH.* Data shown are mean ± SEM from three independent experiments. Statistical significance was assessed using two-way ANOVA. ****$p < 0.0001$, **$p < 0.01$. (b) Effect of siRNA knockdown of key DNA repair pathways on PREMIER. Selected gene targets were knocked down by siRNA targeting. The relative mRNA expression levels were calculated by normalizing to *GAPDH.* n = 3 independent biological replicates. Data are represented as means ± SD. Statistical significance was assessed using one-way ANOVA.

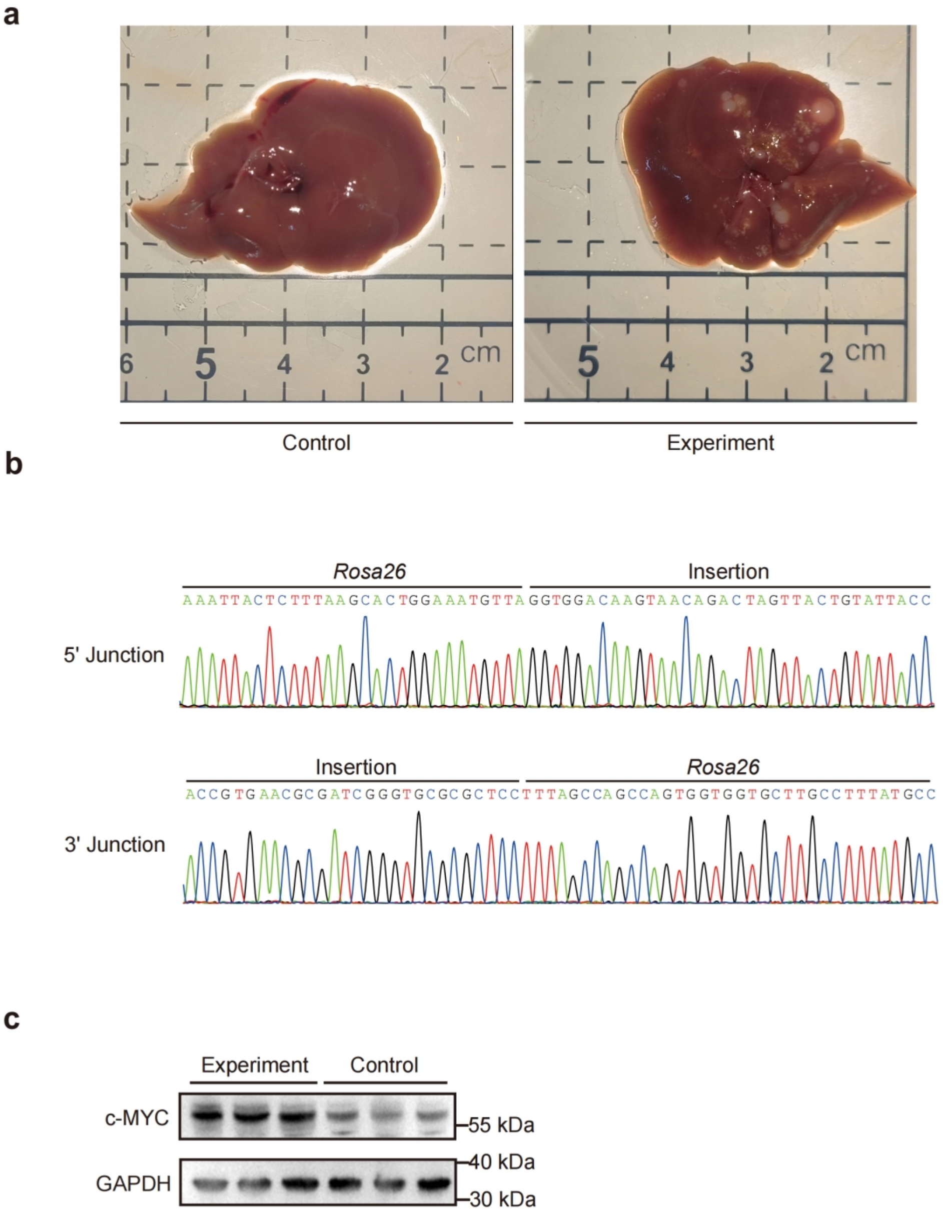

Supplementary Figure. S9. Analysis results of the tumors in the livers of experimental mice.
(a) Presentation of liver tissues from experimental and control groups of mice 20 days post-hydrodynamic injection. (b) Sanger sequencing results of the junctions at both ends of the amplified genomic fragments extracted from tumors in the livers of experimental mice. (c) Expression analysis of c-MYC protein in liver tumors from experimental mice and control mice.

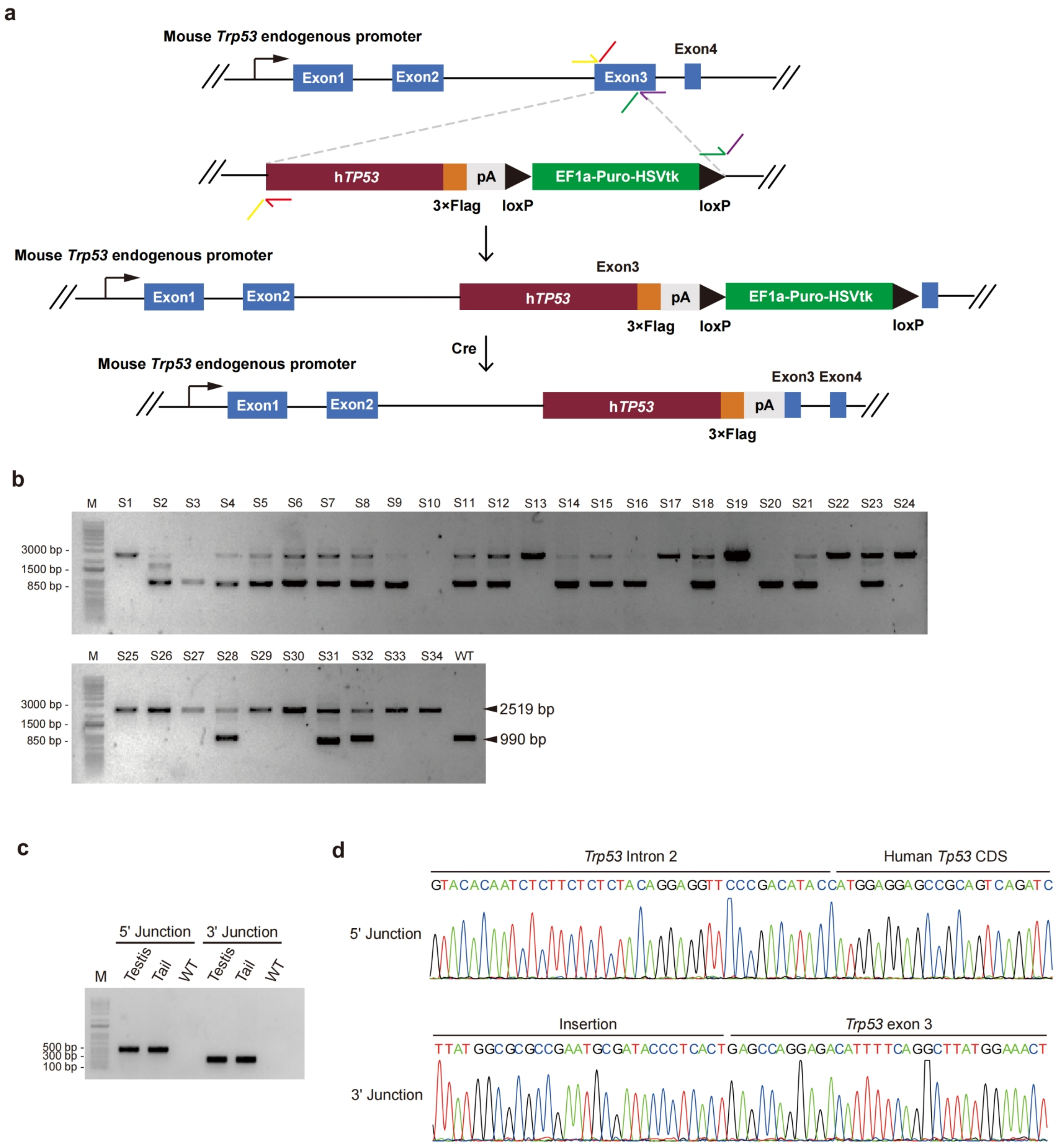
a
Mouse Trp53 endogenous promoter
Exon1
Exon2
Exon3
Exon4
hTP53
pA
EF1a-Puro-HSVtk
3×Flag
loxP
loxP
Cre
b
M
3000 bp -
1500 bp -
850 bp -
2519 bp
990 bp
WT
c
5' Junction
3' Junction
Testis
Tail
WT
500 bp -
300 bp -
100 bp -
d
Trp53 Intron 2
Human Tp53 CDS
GTACACAATCTCTTCTCTACAGGAGGTTCCCGACATACCATGGAGGAGCCGCAGTCAGATC
5' Junction
Insertion
Trp53 exon 3
TTATGGCGCGCCGAATGCGATACCCTCACTGAGCCAGGAGACATTTTCAGGCTTATGGAAACT
3' Junction

Supplementary Figure. S10. Construction and validation of *TP53* humanized chimeric mice

(a) Schematic illustration of replacing the third exon of murine *Trp53* with the human *TP53* CDS using PREMIER.The top diagram illustrates the mouse *Trp53* gene structure, with exons 1 to 4 shown. The middle diagram depicts the donor DNA molecule containing the human *TP53* CDS fused to a 3xFlag tag and a polyA signal. The bottom diagram shows the anticipated outcome after PREMIER-mediated integration, where the human *TP53* CDS replaces exon 3 of the mouse *Trp53* gene, while maintaining the endogenous promoter and the remaining exons. (b) PCR genotyping of genomic DNA extracted from the *TP53* humanized isolated mESC clones. The primers 5'junction-Trp53-F/3'junction-Trp53-R amplify both the wild-type band(990 bp) and the band spanning the *TP53* CDS insertion(2519 bp). (c) PCR genotyping of genomic DNA extracted from the testes and tail of a *TP53* humanized chimeric mouse. The primers 5'junction-Trp53-F/5'junction-TP53-R and 3'junction-bGHpA-F/3'junction-Trp53-R amplify the 5' junction (484 bp) and 3' junction (253 bp) between the humanized *TP53* fragment and the endogenous sequence at the *Trp53* locus, respectively. (d) Sanger sequencing results at the junctions of the inserted fragment in genomic DNA extracted from the testes and tail of a *TP53* humanized chimeric mouse.
